# Brain Computation Is Organized via Power-of-Two-Based Permutation Logic

**DOI:** 10.3389/fnsys.2016.00095

**Published:** 2016-11-15

**Authors:** Kun Xie, Grace E. Fox, Jun Liu, Cheng Lyu, Jason C. Lee, Hui Kuang, Stephanie Jacobs, Meng Li, Tianming Liu, Sen Song, Joe Z. Tsien

**Affiliations:** ^1^Brain and Behavior Discovery Institute and Department of Neurology, Medical College of Georgia, Augusta UniversityAugusta, GA, USA; ^2^The Brain Decoding Center, Banna Biomedical Research Institute, Yunnan Academy of Science and TechnologyYunnan, China; ^3^Department of Computer Science and Brain Imaging Center, University of GeorgiaAthens, GA, USA; ^4^School of Automation, Northwestern Polytechnical UniversityXi’an, China; ^5^McGovern Institute for Brain Research and Center for Brain-Inspired Computing Research, Tsinghua UniversityBeijing, China

**Keywords:** cell assembly, NMDA receptor, cortex, appetitive behavior, computational algorithms, wiring logic, computational logic, social behavior

## Abstract

There is considerable scientific interest in understanding how cell assemblies—the long-presumed computational motif—are organized so that the brain can generate intelligent cognition and flexible behavior. The *Theory of Connectivity* proposes that the origin of intelligence is rooted in a power-of-two-based permutation logic (*N* = 2^*i*^–1), producing specific-to-general cell-assembly architecture capable of generating specific perceptions and memories, as well as generalized knowledge and flexible actions. We show that this power-of-two-based permutation logic is widely used in cortical and subcortical circuits across animal species and is conserved for the processing of a variety of cognitive modalities including appetitive, emotional and social information. However, modulatory neurons, such as dopaminergic (DA) neurons, use a simpler logic despite their distinct subtypes. Interestingly, this specific-to-general permutation logic remained largely intact although NMDA receptors—the synaptic switch for learning and memory—were deleted throughout adulthood, suggesting that the logic is developmentally pre-configured. Moreover, this computational logic is implemented in the cortex via combining a random-connectivity strategy in superficial layers 2/3 with nonrandom organizations in deep layers 5/6. This randomness of layers 2/3 cliques—which preferentially encode specific and low-combinatorial features and project inter-cortically—is ideal for maximizing cross-modality novel pattern-extraction, pattern-discrimination and pattern-categorization using sparse code, consequently explaining why it requires hippocampal offline-consolidation. In contrast, the nonrandomness in layers 5/6—which consists of few specific cliques but a higher portion of more general cliques projecting mostly to subcortical systems—is ideal for feedback-control of motivation, emotion, consciousness and behaviors. These observations suggest that the brain’s basic computational algorithm is indeed organized by the power-of-two-based permutation logic. This simple mathematical logic can account for brain computation across the entire evolutionary spectrum, ranging from the simplest neural networks to the most complex.

## Introduction

Our knowledge regarding specific genes, neuron types and circuitry functions has expanded substantially since Ramón y Cajal pioneered brain research more than a century ago (Mountcastle, 1957; Hubel and Wiesel, 1962; Carraway and Leeman, 1973; Hökfelt et al., 1977; O’keefe and Nadel, 1979; Seeburg et al., 1990; Buck and Axel, 1991; Ramón y Cajal, 1995; Tsien et al., 1996a,b; Tang et al., 1999; Houweling and Brecht, 2008; Klausberger and Somogyi, 2008; Grillner et al., 2013; Kvitsiani et al., 2013; Xu and Südhof, 2013; Alberini and Kandel, 2014; Brichta and Greengard, 2014; Moser et al., 2014; Basu et al., 2016; Tsien, 2016). This pace is likely to accelerate further with recent BRAIN initiatives. However, it has long been recognized that there is a need to establish the basic computational frameworks that may underlie the brain’s functions (Grillner, 2006; Tsien, 2007; Brenner and Sejnowski, 2011; Shanahan, 2012; Budd and Kisvarday, 2013; Marcus et al., 2014; Geman and Geman, 2016). Imagine if all component parts were made available; what should be the unifying mathematical principles that evolution has adhered to in constructing brains in such a way as to be capable of intelligent cognition and adaptive behavior be?

Clearly, this is a daunting question. The human brain is estimated to have approximately 86 billion neurons (Herculano-Houzel, 2009), and each neuron has tens of thousands of synapses (Andersen, 1990), leading to over one hundred trillion synaptic connections. On top of this astronomical complexity, one needs to map each connection or neuron to a given stimulus, yet possible numbers of stimuli that can be used are infinite given the complex, ever-changing nature of the world we live in. Adding yet another layer of complexity to this seemingly hopeless situation are the well-known variations in the number of neurons, axonal/dendritic branches and synapses—not only over the course of development and aging, but also across individual brains and animal species. As such, the unifying mathematical principle upon which evolution constructs the brain’s basic wiring and computational logic represents one of the top most difficult and unsolved meta-problems in neuroscience (Adolphs, 2015; Geman and Geman, 2016).

Recently, we have taken an alternative “*thought-experiment*” approach to this question (Tsien, 2015a,b; Li et al., 2016). We reasoned that the essence of intelligence lies in the brain’s ability to discover specific features and generalized knowledge from a world full of uncertainties and infinite possibilities; therefore, our search for the brain’s computational logic can be reduced to the question of how neurons should be connected in such a way that would inherently afford the brain to discover various patterns and conceptual knowledge.

One useful concept in pursuing this line of reasoning is *cell assembly*, a term coined by Hebb (1949) to describe the supposed computational building block or computational primitive in the brain (Hebb, 1949). This notion has attracted keen interest, especially with emerging large-scale recording techniques (Nicolelis et al., 1997; Kudrimoti et al., 1999; Lin et al., 2006b; Maurer et al., 2006; Tsien, 2007; Buzsáki, 2010; Tsien et al., 2013). Hebbian cell assembly was postulated to be comprised of a group of neurons with strong excitatory connections that are formed after learning. Once a subset of its cells is later stimulated (i.e., by cues to recall memory), the assembly would be activated as a whole to represent percepts or concepts (Hebb, 1949; Wallace and Kerr, 2010). While this terminology capitalizes on the presumed building block in the brain, the wiring and computational logic of such a computational motif remain a mystery.

There is a long list of questions that one can ask: How do principal cells within a cell assembly organize themselves? How can such a logic of connectivity enable pattern-separation and pattern-generalization? How should the size of a cell assembly be defined? In an abstract sense, is there a mathematical principle governing the unifying computational algorithm conserved across various neural circuits despite their different anatomical features? Furthermore, to what degree is such cell-assembly logic dependent on learning in adulthood or largely genetically programmed during brain development? Finally, how is the computational logic implemented in the cortex so that it can be repeatedly utilized as a basic motif via surface expansion, leading to greater intelligence?

### The *Theory of Connectivity* and its Predictions: A permutation-Based Wiring Logic To Cover Every Possibility

**Figure 1:**
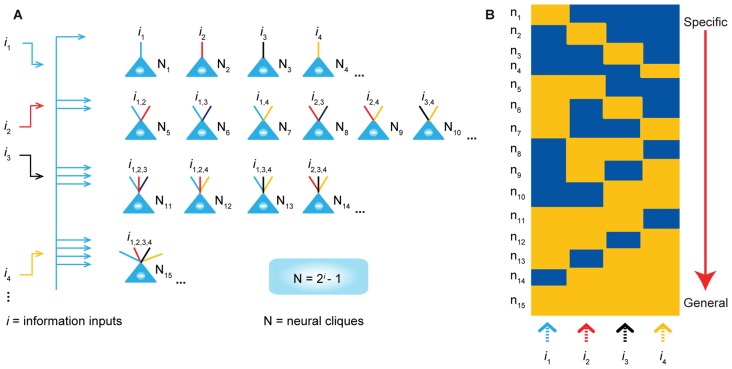
**The power-of-two based permutation logic for governing the specific-to-general wiring and computational logic of cell assemblies. (A)** The equation defines the size of a cell assembly; the numbers of neural cliques within a cell assembly. By following the permutation equation of *N* = 2^i^–1, the cell-assembly motif exemplified here consists of 15 distinct neural cliques (*N*_1_…_15_), which cover all possible connectivity patterns in order to process four distinct inputs (*i*_1_, *i*_2_, *i*_3_, *i*_4_). The specific-to-general neural cliques shown in this subpanel illustrate the logic for wiring non-recurrent networks (e.g., the hippocampal CA1).** (B)** Schematic “bar-code” illustrates the specific-to-general cell-assembly activation patterns, which can be measured by electrodes or imaging techniques, from the 15 distinct neural cliques (N_1–15_), processing four distinct inputs (*i*_1_, *i*_2_, *i*_3_, *i*_4_). The orange color represents the stimulus-triggered activation above the baseline state (in blue). The arrow on the right side illustrates the number of distinct neural cliques exhibiting specific, sub-combinatorial, as well as generalized, responsiveness. The cartoon illustration was adopted from Tsien, TINS, 2015. Specific neural cliques encode specific features, whereas various permutation rule-based neural cliques encode various convergent patterns, representing relational memories and generalized concepts.

To explore these questions, we have put forth the *Theory of Connectivity* that proposes a rather simple mathematical rule in organizing the microarchitecture of cell assemblies into the specific-to-general computational primitives that would readily enable knowledge and adaptive behaviors to emerge in the brain (Tsien, 2015a,b; Li et al., 2016). The theory specifies that within each computational building block, termed “functional connectivity motif” (FCM), the total number of *principal projection-cell* *cliques* with distinct inputs should follow the power-of-two-based permutation equation of *N* = 2^i^**–1** (*N* is the number of distinct neural cliques that can cover all possible *permutations* and *combinations* of specific-to-general input patterns, whereas *i* is the number of distinct information inputs; Figure 1). As such, each FCM consists of principal projection neuron cliques receiving specific inputs, as well as other principal projection neuron cliques receiving progressively more convergent inputs that systematically cover every possible pattern using the power-of-two-based permutation logic (Figure 1A).

In other words, an FCM is made of neural-clique assemblies arranged from specific input-coding principal cell assemblies to sub-combinatorial and to general responsive cell assemblies. The specific neural cliques extract unique features about perspective stimuli (from external environments and/or internal sources), whereas the sub-general and general neural cliques categorically extract all possible combinational patterns. Cognitively speaking, specific and sub-general cell cliques encode specific memories or actions for pattern-discrimination and categorization, respectively, whereas higher combinational or generalized neural cliques discover general patterns for pattern-generalization corresponding to semantic memories, categorical knowledge, general intent and motor instruction. In essence, this power-of-two-based permutation logic intrinsically enables each FCM to cover every mathematical possibility of connectivity patterns in a specific-to-general manner (Tsien, 2015a,b).

Due to the inherent structure-function relationship, this power-of-two permutation-based wiring logic can be functionally detected by neural recording techniques in the form of specific-to-general cell assembly activation patterns (Figure 1B). For example, the theory predicts that by providing four distinct stimulus inputs (i.e., *i* = 4), one should observe all 15 excitatory cell cliques (*N*_1–15_) in relevant brain regions that exhibit specific-to-general coding properties.

Importantly, this *Theory of Connectivity* has offered six testable predictions: (1) Cognitive universality—This power-of-two-based computational logic should be used to process various cognitions across a wider range of modalities—including appetitive, emotional and social information; (2) Anatomical prevalence—This logic should be prevalent across many cortical and subcortical circuits, regardless of their macroscopic and microscopic variations; (3) Modulatory neurons, such as dopamine (DA) neurons, use a different logic; (4) The specific-to-general organization should be developmentally pre-configured, rather than to be formed after learning in adulthood; (5) This computational logic is implemented in the cortex vertically via the differential assignment of specific-to-general cliques to distinct laminar layers. This vertically implemented FCM has the advantage to be readily replicated via horizontal surface expansion (rather than via expansion of cortical thickness); and (6) Species conservancy—The proposed computational logic is evolutionarily conserved across the brains of different animal species. Here, we describe a series of experiments in testing these six predictions derived from the power-of-two permutation-based computational logic.

## Materials and Methods

### Ethics Statement

All animal work described in the study was carried out in accordance with the guidelines established by the National Institutes of Health regarding the care and use of animals for experimental procedures and was approved by the Institutional Animal Care and Use Committee at the Medical College of Georgia at Augusta University (Approval AUP number: BR10–12–392) and Banna Biomedical Research Institute of Yunnan Academy of Science and Technology (BBRI#102).

### Construction of Tetrode Headstages and Animal Surgery

Tetrodes and headstages were constructed using the procedures as we have previously described (Lin et al., 2006a; Xie et al., 2016). To construct tetrodes, a folded piece consisting of four wires (90% platinum, 10% iridium, 13 μm, California Fine Wire Company, Grover Beach, CA, USA) was twisted together using a manual turning device and soldered with a low-intensity heat source (variable temperature heat gun 8977020, Milwaukee, Brookfield, WI, USA) for 6 s. The impedances of the tetrodes were measured with an electrode impedance tester (Model IMP-1, Bak Electronics, Umatilla, FL, USA) to detect any faulty connections, and our tetrodes were typically between 0.7 MΩ and 1 MΩ. The insulation was removed by moving the tips of the free ends of the tetrodes over an open flame for approximately 3 s. The tetrodes were then placed into appropriate polyimide tubes. The recording ends of the tetrodes were cut differentially (Vannas spring scissors −3 mm cutting edge, Fine Science Tools, Foster City, CA, USA) according to the different depths of the recording sites. This ensures that only tetrodes, but not the surrounding polyimide tubes, were inserted into the brain tissue, thereby minimizing the tissue damage.

We employed adjustable 128-channel tetrode microdrives to target the basolateral amygdala (BLA; *n* = 9 WT mice), medial amygdala (MeA; *n* = 9 WT mice), anterior cingulate cortex (ACC; *n* = 5 WT mice), retrosplenial cortex (RSC; *n* = 9 WT mice) and CA1 (*n* = 5 WT mice) bilaterally with 64 channels per hemisphere (Lin et al., 2005, 2006a). Stereotaxic coordinates were as follows: for the mouse BLA, 1.7 mm posterior to bregma, 3.5 mm lateral, −4.0 mm ventral to the brain surface; for the mouse MeA: −1.70 mm anterior-posterior (AP), ± 2.1 mm mediolateral (ML), −5.1 mm dorsoventral (DV); for the mouse ACC: +0.50 mm AP, ± 0.5 mm ML, −1.75 mm DV; for the mouse RSC: −2.5 mm AP, ±0.5 mm ML, −0.8 mm DV; and for recording in the prelimbic cortex (PrL) of the Golden Syrian hamster, the stereotaxic coordinate was +3.50 mm AP, ± 0.7 mm ML, −4.0 mm DV.

For recording in cortical layers 2 and 3 (L2/3) of the RSC, nine mice were used (eight mice were implanted with 32-channel tetrodes and one mouse with a 64-channel tetrode array). For recording in cortical layers 5 and 6 (L5/6) of RSC, 11 mice were used (seven mice were implanted with 32-channels, three mice with 64 channels bilaterally and one mouse with 128 channels bilaterally). For recording from the mouse CA1, the electrode bundles were positioned above the bilateral dorsal hippocampi (2.0 mm lateral to the bregma and 2.3 posterior to the bregma on both the right and left sides). To record in the ILA region, we used 64-channel tetrodes with 32 channels per hemisphere (*n* = 5 mice). The electrode bundles were positioned above the ILA (1.70 mm anterior to bregma and 0.5 mm lateral on each side to a depth of 2.0 mm). For recording in the mouse ventral tegmental area (VTA), we used 64-channel tetrodes with 32 channels targeting each side or a single 32-channel tetrode bundle for a single side (3.4 mm posterior to bregma, 0.5 mm lateral and −3.8 mm to −4.0 mm ventral to the brain surface).

Male wild-type (WT) and KO mice (6–8 months old) or adult male hamsters (3–4 months old) were moved from home cages housed in the LAS facility to the holding area next to the chronic recording rooms in the laboratory and stayed in a large plastic bucket (20 inches in diameter and 16 inch height, Walmart) per mouse as their homes with access to water and food for a week prior to surgery. During this period, the animals were also handled daily to minimize the potential stress from human interaction. On the day of the surgery, the animal was given an intraperitoneal injection of 60 mg/kg ketamine (Bedford Laboratories, Bedford, OH, USA) and 4 mg/kg Domitor (Pfizer, New York, NY, USA) prior to the surgery. The head of the animal was secured in a stereotaxic apparatus, and an ocular lubricant was used to cover the eyes. The hair above the surgery sites was removed, and Betadine solution was applied to the surface of the scalp. An incision was then made along the midline of the skull. Hydrogen peroxide (3% solution, Fisher Scientific) was placed onto the surface of the skull so that bregma could be visualized. The correct positions for implantation were then measured and marked. For fixing the microdrive headstage, four holes for screws (B002SG89S4, Amazon, Seattle, WA, USA) were drilled on the opposing side of the skull and, subsequently, the screws were placed in these holes with reference wires being secured to two of the head screws. Craniotomies for the tetrode arrays were then drilled, and the dura mater was carefully removed. After the electrodes were inserted and tetrodes were secured to the fiberglass base, and the reference wires from the connector-pin arrays were soldered such that there would be a continuous circuit between the ground wires from the head screws and those from the connecter-pin arrays. Finally, the connector-pin array was coated with epoxy. Aluminum foil was used to surround the entire headstage to aid in protection and to reduce noise during recordings. The animals were then awoken with an injection of 2.5 mg/kg Antisedan. The animals were allowed to recover post-surgery at least for 3–5 days before recording began. Then, the electrode bundles targeting the BLA, VTA, MeA, and hippocampal CA1 region were slowly advanced over several days in small daily increments. For the cortical sites, tetrodes were advanced usually only once or twice in a small increment.

### Behavioral Paradigm and *In Vivo* Recording

After surgery, the animals were handled for another 5–10 days while electrodes were advanced to the recording sites for obtaining maximizing neural units. On the day(s) of behavioral experiments, we recorded the neural ensemble activity in freely behaving mice in a clean home bucket for at least 30 min as baseline. For appetitive experiments, various foods (sugar pellets, rice, rodent diet pellets and milk droplets made from Instant nonfat dry powder) were delivered to the small petri dish located in the clean home bucket to minimize emotional effects due to changes in environment. Seven pellets or milk droplets were delivered for each food type with a 15–30 s time-interval after consumption. The order of delivery was randomized by using different delivery orders for different animals. We noted that mice typically consumed the food within 10–20 s. The time intervals between the different foods were 5–10 min. The recordings were continued for an additional 30 min after all the appetitive experiments were completed. The experiments were videotaped by a camera placed above the recording chamber.

For social interaction experiments, we implemented 64-channels in the right hemisphere MeA in four mice and 128-channels bilaterally in the MeA in five mice. These mice were single-cage housed for at least for 1 month before surgery. One week prior to surgery, the mice were transferred to the large home buckets (46-cm in diameter) located in the recording room and maintained single-cage housed and socially isolated until the social interaction experiments. On that day, the recording mouse was first placed in the clean home bucket for 30 min (this also minimized the potential influences from the change in recording environments) and recorded as the resting baseline state before a social stimulus mouse (either male or female) was introduced. To reduce mating behavior, we used anestrus females. Two mice were allowed to freely interact for 10 min and then the stimulus mouse was removed with a 15-min time interval before a second mouse (male or female) was introduced. The sex of the stimulus mice was balanced across the trials. Social behaviors during these interactions were videotaped. The most prominent social interactions were the face-to-face interactions and face-to-anogenital interactions. To facilitate peri-event spike raster and histogram analyses, we screened the videotapes and marked social interaction when face-to-face or face-to-anogenital interactions occurred at 1-cm distance as time zero. In some cases, the animals engaged in dynamic interaction from the anogenital area to face or vice versa within 1 s. These trials were excluded from peri-event spike raster analysis to minimize the carry-over effects.

For fearful-event stimulation experiments, the animal was introduced to the airpuff chamber (a clean large home bucket), foot-shock chamber, earthquake chamber and drop boxes. Baseline activities were recorded for at least 3–5 min prior to fearful stimulation. Four distinct fearful episodic events were introduced to the mice in a fixed sequence: (1) Airpuff - a sudden airpuff was delivered to the animal’s back (10 p.s.i., 400 ms) via an air tube; (2) Earthquake-like shake - the mouse was placed in a small chamber (4" diameter and 6"H circular chamber) fixed on top of a vortex mixer and shaken at 300 rpm for 400 ms (Lin et al., 2005); (3) Free-fall in the elevator - the animal was placed inside a small box (3" x 3" x 5"H) and dropped from an 11-cm height (a cushion which was made from a crumbled table cloth was used to dampen the fall and to stop the bouncing effect), and (4) Fearful-conditioning foot-shock. The fear conditioning chamber was a square chamber (10" × 10" × 15"H) with a 24 bar, shock-grid floor (Zhang et al., 2013). In a subset of experiments, acoustic startle produced by clapping two metal boxes (100 db, 300 ms in duration) was also used. Animals were placed into the shock chamber for 3–5 min and received the unconditioned foot-shock stimulus (60 Hz phasic 300-ms foot-shock at 0.75 mA) for a total of seven times and an inter-trial time interval between 1–3 min. These episodic stimuli are fearful as evidenced by physiological indications, including a rapid increase in heart rates and reduced heart rate variability (Liu et al., 2013, 2014). To facilitate peri-event spike histogram analysis, each fearful event was repeated 7–10 times. To maintain the consistency of stimulus inputs yet minimizing the possible prediction of upcoming stimuli, the episodic stimuli were triggered by a computer and delivered at randomized intervals within 1–2 min. We also introduced 5- to 10-min intervals (resting in home buckets) between switching from one type of fearful event session to another session of a different type of stimulus while briefly placing the mice in the home buckets. After the completion of all episodic event sessions, the mice were placed back into the home buckets.

At the end of the chronic recording experiments, the animals were anesthetized and a small amount of current was applied to the recording electrodes in order to mark the positions of the tetrode bundles. The actual electrode positions were confirmed by histological Nissl staining using 1% cresyl echt violet. In some experiments, the electrode tips were dipped in fluorescent Neuro-Dil (Neuro-Dil, #60016, Red oily solid color, from Biotium, Inc.) prior to surgery insertion, which can then reveal the electrode track under a fluorescent microscope. 4′, 6-diamidino-2-phenylindole (DAPI) staining was used for the counter-staining of the nuclear DNA of the brain cells. Images were collected using a Zeiss 780 Upright Confocal microscope. The stability of the *in vivo* recordings was judged by waveforms at the beginning of, during and after the experiments. Only stable units were included for further analysis.

### Data Processing and Spike Sorting

The neuronal activity was recorded by a Plexon multi-channel acquisition processor system (filtered at 250–8000 Hz; digitized at 40 kHz), and waveforms were collected using 56 points with 1400 μsec time width. The recorded spike activities from various brain regions were processed in the manner also previously described (Lin et al., 2006a; Zhang et al., 2013), and then sorted using the MClust 3.5 program^1^. First, the recorded data were filed in Plexon system format (*.plx). Before spike sorting, the artifact waveforms were removed and the spike waveform minima were aligned using the Offline Sorter 3.3.5 software^2^, (Dallas, TX, USA). It should be noted that foot-shock did introduce artifacts, but could be easily removed during this spike sorting step because spike waveforms are distinct from electrical noise. The aligned data were then saved as files in a Neuralynx System format (*.ntt). Next, the MClust 3.5 program was used to isolate different spiking units. Only units with less than 0.1% in spike intervals within a 1-ms refractory period and clear boundaries, as judged by an Isolation-Distance calculation >15, were included in the present analysis.

For the datasets recorded from ACC, CA1, PrL, RSC and ILA, well-isolated units were classified as either putative excitatory principal cells or inhibitory interneurons based on three characteristic features of spike waveform—namely, trough-to-peak width, the second principal component of the spike waveform (PC2), and the first derivative of the energy (EnergyD1 in MClust). Automated clustering of excitatory principal cells and inhibitory interneurons was performed by *k*-means method.

Because the features PC2 and EnergyD1 provided poor differentiate power in BLA datasets, we employed a novel set of spike waveform features for describing the spike waveforms of neuron firing in BLA, including trough-to-peak width, half-width after trough and the differential integral of area shape Δ*A*_(after peak)_. Half-width after trough was defined as the width between the points when the waveform raised to or fell from half-height of the peak. Δ*A*_(after peak)_ was defined as the area between the waveform and the line segment of peak and the last point of the waveform. The feature Δ*A*_(after peak)_ could be positive or negative, depending on the shape of the waveform after the peak. The *k-means* method was employed to achieve automated cell-type clustering, and we found that the vast majority of the units recorded from BLA were putative excitatory principal cells (~86.4%).

### Construction of Optrodes and Light-Stimulation Protocol

For the optrodes targeting the BLA or VTA bilaterally, a square-shaped arrangement of polyimide tubes per bundle was used for 32 channels/8 tetrodes per hemisphere. For optical fiber, cladding was removed from the two 200-μm core, 037 NA standard, hard-cladding, multimode fiber (ThorLabs), and the optical fibers were placed 1-mm apart in a microdrive base for bilateral targeting of the VTA or BLA using a total of 32 channels/8 tetrodes. Eight tetrodes were threaded into separate polyimide tubes and placed adjacent to each optical fiber to create an optrode bundle with the tip of the optical fibers 600-μm above the recording tetrodes. Tetrodes were then inserted into eight of the polyimide tubes, leaving an empty tube at each corner of the square. This arrangement would allow the tetrodes to be reached effectively by the light-stimulation from the fiber. After each tetrode was correctly positioned, the wires were glued to the polyimide tubes. The free ends of each tetrode were wrapped around the 36 pin-connector array and were individually soldered to their respective pins. The recording tips of the tetrodes were cut so that they would protrude past the fiber 300–500 μm.

Prior to stimulation, PM100D (ThorLabs) was used to measure the light intensity. Optical fibers were connected to a blue laser (473 nm, diode-pumped solid-state, Shanghai Dream Lasers Technology Co.). Trains of 10-Hz stimulations (10 ms per pulse, 20 pulses per train) were delivered to the site in 10 trials using a PulsemasterA300. To confirm the identity of pyramidal cells that were based on waveform characteristics, we recorded from the BLA of two double transgenic mice (Tg-CaMKII-Cre crossed with Tg-Ai32-ChR2). To verify DA neurons, we used a combination of pharmacological and optogenetic methods that we previously published (Wang and Tsien, 2011). DA neurons were identified initially by decreased firing upon injection of D2 agonist apomorphine (1 mg/kg, i.p.). To label DA neurons using optogenetics, we injected adeno-associated virus particles (UNC viral core facility), coding the floxed channelrhodopsin-2 (ChR2) and green fluorescent protein (GFP), into the VTA region of a DAT-Cre mouse. The animals were allowed to recover for 3–4 weeks before recording. Optogenetic identification of putative pyramidal cells or DA neurons was based on the comparison between waveforms before optical stimulation and those triggered by blue laser during the light-stimulation. Waveforms were judged to be identical if the correlation coefficient was measured to be higher than 0.9. The latencies between the light-stimulation and blue-light-induced spikes were calculated by peri-event histogram.

### Characterization of Principal Unit Responses

To determine whether a recorded unit was responsive to a given stimulus, we used the stimulus time points as time zeros to calculate a peri-event histogram using a 500 ms-bin size for appetitive stimulation, a 200 ms-bin size for social interaction, and a 100 ms-bin size for fearful stimuli. The neural activities before stimulus were used as a baseline to determine confidence intervals (10-s for appetitive experiments, 3-s for social recognition, and 2-s for fearful stimulation). To assess significant changes in firing, we used 95%, 99.9%, 99.999%, confident intervals and the duration longer than five bins, three bins, and two bins for a given category of stimulation, respectively, then it is considered a significant neuronal response to the stimulus. These criteria are determined empirically. To facilitate the comparison between units that exhibit different increases/decreases over baseline activities, we used the transformation *R*_i_ = (*f*_resp, i_ − *f*_pre, i_)/(*f*_0_ + *f*_resp, i_) Here, *f*_resp, i_ represents the average firing rate during the detected neuronal response after the stimuli *i*. *f*_pre_ is the average firing rate during baseline before the stimuli *i*, and *f*_0_ is the averaged basal firing rates of all isolated units in a given region (typically in the range of 0.5~3.5 Hz). Note that this transformation allows for uniform quantification of the significant changes in firing patterns for units with both low- and high-baseline firing rates.

For measuring the population response significance, neural activity was calculated by comparing the firing rate after stimulus onset (in 500 ms-, 200 ms- or 100 ms-bin size for appetitive, social, or fearful stimuli, respectively) with the firing rate recorded during the baseline periods (10-s before appetitive experiments, 3-s before social recognition, and 2-s before fearful stimulation) using a *Z*-score transformation. *Z-score* values were calculated by subtracting the average baseline firing rate established over the defined duration preceding stimulus onset from individual raw values then by dividing the difference by the baseline standard deviation.

### Hierarchical Categorical Clustering

Hierarchical clustering methods were used to investigate the stimulus responses of the overall population of the simultaneously recorded units. The procedure was similar to the one previously described (Lin et al., 2005; Zhang et al., 2013). This analysis was performed on a transformed neuronal response: *T* = log(1+|*R*|). Here, *R* is a *n* × *m* matrix representing the neuronal responses of *n* units during *m* stimulus, and | | denotes the absolute value. An agglomerative hierarchical cluster tree was created from the standardized Euclidean distances. Then, a categorical sorting was applied to facilitate the visualization. That is, units were sorted by the number of stimuli to which these units responded. After the categorical sorting, the units which were non-responsive were put on the top of the matrix, followed by the units responding to a specific event, and the units responding generally to the most number of types of stimulus events were located at the bottom of the matrix.

### Assessing Nonrandomness in Distribution Patterns of Neural Cliques

In order to test if the response patterns of the recorded ensembles deviated from random distribution, we constructed a null (independent random) model which assumed that each neuron in the given region has an independent response probability to a given pattern, and the response probabilities of this neuron to different stimuli can be different. In other words, each event would randomly activate a subset of the neurons with a different average ensemble size, and the activation patterns of different stimuli are independently chosen and do not interact. Accordingly, we normalized the observed histogram of counts for 16 clique response-types to four different stimuli (example shown below) by the total number of counts to obtain the response pattern distributions denoted as *p*_i_(*E*_j_), where i goes from 1–16, and j from 1–4, and E_j_ can be 0 or 1. Example histogram for ACC WT mice:

#1 clique (0, 0, 0, 0): 114 cells (non-responsive units)

#2 clique (1, 0, 0, 0): 35 cells (responded to the first stimulus)

#3 clique (0, 1, 0, 0): 25 cells (responded to the second stimulus)

#4 clique (0, 0, 1, 0): 13 cells (responded to the third stimulus)

#5 clique (0, 0, 0, 1): 35 cells (responded to the fourth stimulus)

#6 clique (1, 1, 0, 0): 23 cells (responded to the first and second stimuli)

#7 clique (1, 0, 1, 0): 15 cells (responded to the first and third stimuli)

#8 clique (0, 1, 1, 0): 13 cells (responded to the second and third stimuli)

#9 clique (1, 0, 0, 1): 21 cells (responded to the first and fourth stimuli)

#10 clique (0, 1, 0, 1): 13 cells (responded to the second and fourth stimuli)

#11 clique (0, 0, 1, 1): 14 cells (responded to the third and fourth stimuli)

#12 clique (1, 1, 1, 0): 37 cells (responded to the first, second, and third stimuli)

#13 clique (1, 1, 0, 1): 19 cells (responded to the first, second, and fourth stimuli)

#14 clique (1, 0, 1, 1): 17 cells (responded to the first, third, and fourth stimuli)

#15 clique (0, 1, 1, 1): 31 cells (responded to the second, third, and fourth stimuli)

#16 clique (1, 1, 1, 1): 95 cells (responded to all four stimuli)

The probability of generating the combined pattern is the product of individual independent probabilities, i.e., *p_i_* (*E*_1_ = 0, *E*_2_ =1, *E*_3_ =0, *E*_4_ = 0) = *p*(*E*_1_ = 0) × *p*(*E*_2_ = 1) × *p*(*E*_3_ = 0) × *p*(*E*_4_ = 0). The independent probabilities can be obtained by summing all *p*_i_*s* where a given event occurs. For example, *p*(*E*_1_ = 0) can be obtained by summing all *p*_i_*s* with *E*_1_ = 0, regardless of the value of the other variables. To generate the error bars, we used a resampling procedure by running 1000 Monte-Carlo simulations. In each simulation, we assigned each data point to one of those 16-clique patterns by drawing a random number weighted by the probability of the occurrence of each pattern. The 5% and 95% values were plotted on the graphs, and the *p* value for the probability of a given pattern being significantly different from the actual distribution was assessed at a level of *p* < 0.05 after dividing the *p* value by 16 to reflect the Bonferroni multiple hypothesis testing correction.

## Results

### The Power-of-Two-Based Permutation Logic for Processing Food Experiences

We employed large-scale *in vivo* tetrode arrays (Lin et al., 2006a) to measure activation patterns of large numbers of neurons in the appetitive neural circuit—namely, the BLA (Everitt et al., 2003; Fernando et al., 2013), while mice freely consumed four different foods (rodent-diet pellets, sugar pellets, rice and milk droplets). Because our *Theory of Connectivity* concerns the coding patterns of principal-projection neurons, we limited our present analysis to putative excitatory neurons (for classification of putative pyramidal cells and fast-spiking interneurons, as well as their stability during experiments, please see Supplementary Figures S1A–1C). To avoid possible training-induced changes in coding patterns, we only used the datasets collected from the very first food-encounter experiments during which the mice consumed seven sugar pellets, seven rice and seven condensed-milk droplets for the first time, in addition to seven rodent diet pellets (which lab animals routinely ate).

**Figure 2:**
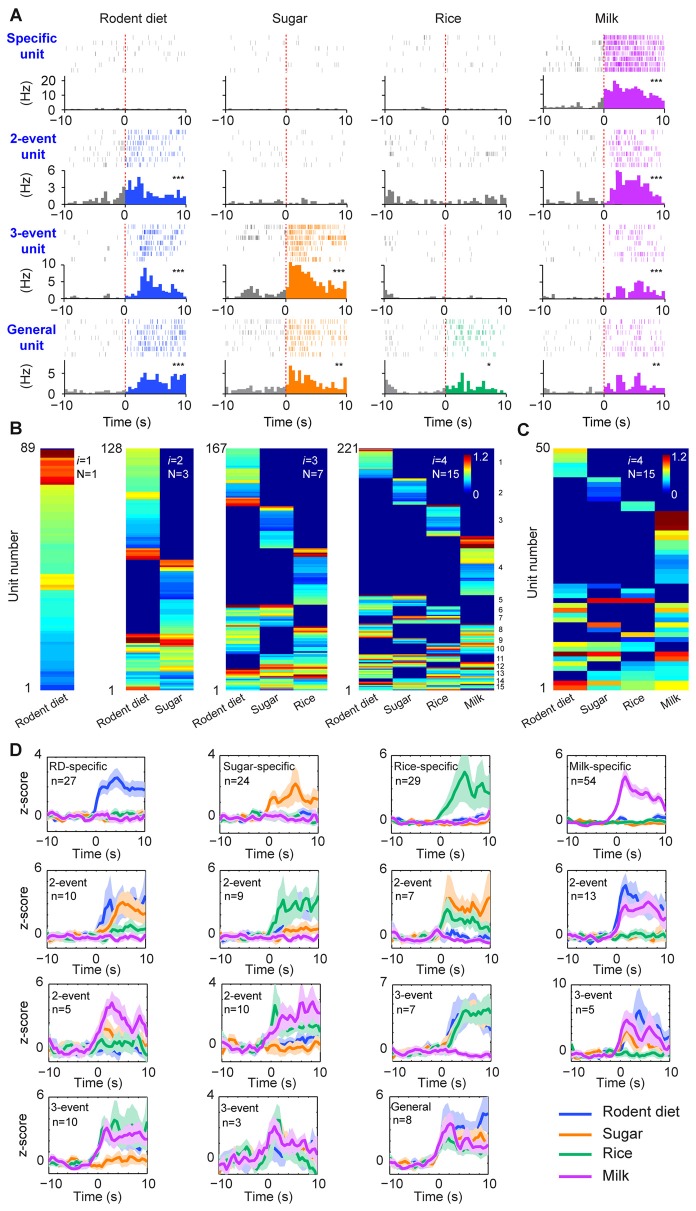
**The permutation-based logic in the basolateral amygdala (BLA) cell assemblies for processing food experiences. (A)** Examples of pyramidal cells with specific to general responsiveness. The top row shows a cell selectively responded to milk; A representative two-event cell responding to rodent diet biscuit (RD) and milk (the second row); A three-event cell responding to biscuit, sugar and milk (the third row); A pyramidal cell responding to all four types of foods (bottom row). Peri-event raster (upper subgraph) and peri-event histograms (lower subgraph) are presented. *P < 0.05, **P < 0.01, ***P < 0.001, Wilcoxon signed-rank test. **(B)** BLA pyramidal cells followed the power-of-two-based permutation rule to process appetitive information (*n* = 9 mice, 221 responsive pyramidal cells). As *i* was increased from 1, 2, 3, to 4, hierarchical clustering plots show the emergence of distinct neural cliques from 1, 3, 7, to 15. The *Y-axis* lists the number of responsive putative pyramidal cells. Color scale bars indicate the logarithm-transformed responsiveness of putative pyramidal units (10-s post-stimulation) averaged over seven trials. It typically took 5~10 s for mice to consume each pellet or milk. **(C)** The specific-to-general assembly logic was also present in the simultaneously recorded BLA pyramidal cells from a single mouse (50 out of 110 pyramidal cells were responsive to foods) in the very first recording session. In this mouse, the food order was presented as follows: seven rodent biscuits, seven sugar pellets, seven rice, followed by seven milk droplets. **(D)** *Z-score* plots show population responses of each of the 15 distinct neural cliques in the BLA to various food experiences. The number of pyramidal cells belonging to each clique is listed in each subplot. Color lines (blue, orange, green, purple) indicate a given food type.

A total of 794 units were recorded from the BLA in nine WT mice, and 527 putative pyramidal units met the above criteria and were used for peri-event spike raster histogram analysis. Interestingly, we found that putative pyramidal cells exhibited a variety of event-response selectivity, including the general-responsive cells that increased their firings to all four types of foods, the combination of two or three types of appetitive stimuli, or those specific units responding only to one type of food (Figure 2A). The diverse responsiveness among the putative pyramidal cell population was further confirmed by optrode recording from Tg-CaMKII-Cre/Ai32-ChR2 double transgenic mice. These computationally classified pyramidal cells were activated by blue light and they indeed exhibited specific, combinatorial, or general responses to appetitive stimulations (Supplementary Figures S1D,F).

From a neural classifier’s perspective, this was an emergent process of pattern self-discovery as the animals experienced different foods over the meal session. For example, of these 527 BLA principal cells, 89 of the units (16.89%) increased firings to rodent diet (Figure 2B, the first heat map on the left). When the responses to rodent diet and sugar pellets were analyzed, a total of 128 pyramidal cells responded with three distinct neural cliques (Figure 2B, the second heat map) —30 units increased firing to both rodent diet and sugar, indicating that these neurons received converged inputs; whereas 59 units reacted specifically to rodent diet, and 39 units to sugar only. As neuronal responses to rice were included in data analysis, a total of 167 units were found to be involved in encoding these three food experiences, with a total of seven specific-to-general combinatorial cliques manifested (Figure 2B, the third heat map). When milk responses were included, a total of 15 cell cliques has emerged. These 15 pyramidal cell cliques precisely followed the power-of-two-based permutation logic (***N*** = 2^4^− 1 = 15) which covered every mathematical possibility required to process four distinct food experiences (Figure 2B, the fourth heat map). Importantly, the emergence of 15 distinct neural cliques was also observed in a single-mouse dataset (Figure 2C), demonstrating that this computational logic indeed operates within the local motif. The diverse response patterns of these 15 neural cliques was further confirmed by the *Z-score* analysis plot of pooled population datasets collected from multiple mice (*n* = 9, each animal was subjected to a different food order; Figure 2D). Therefore, the BLA pyramidal cells followed the specific-to-general permutation logic to extract all possible patterns, enabling discrimination, categorization, and generalization of various food experiences.

### The Power-of-Two-Based Logic for Processing Social Interactions

**Figure 3:**
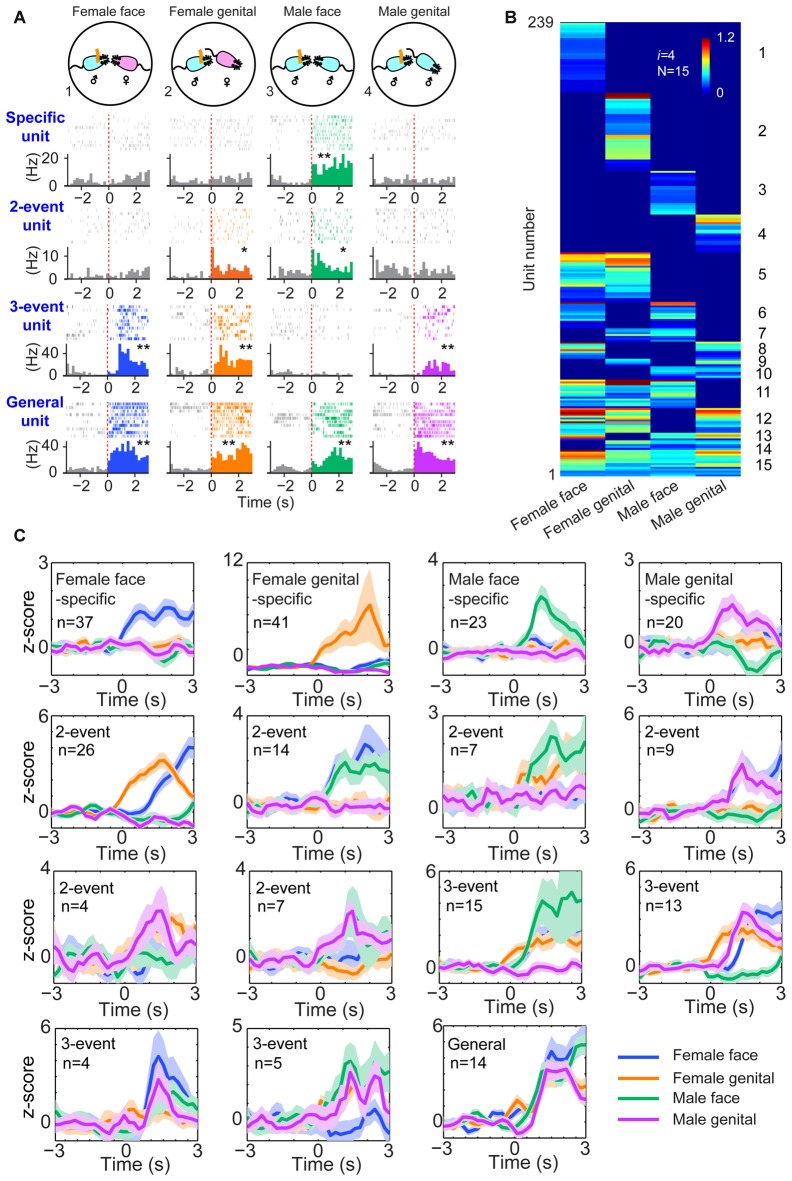
**The power-of-two-based logic in the medial amygdala (MeA) cell assemblies for processing social information. (A)** Four distinct social recognitions: female face, female anogenital area, male face and male anogenital area. Examples of specific-to-general responsive pyramidal cells. *P < 0.05, **P < 0.01, Wilcoxon signed-rank test. **(B)** Hierarchical clustering plot shows the existence of 15 distinct neural cliques in the MeA. The datasets were pooled from nine mice (239 responsive units). The *Y-axis* lists the number of responsive units. Color scale bars indicate the scaled responsiveness of MeA units. **(C)** *Z-score* plots show population responses of the 15 distinct neural cliques in the MeA to four distinct social interactions. The number of cells belonging to a given clique is indicated inside each plot. Colored lines (blue, orange, green, purple) indicate different social interaction types.

Next, we examined whether this power-of-two-based computational logic is employed in processing social recognition. The MeA is known to be part of the social-behavioral neural network (Petrulis, 2009; Gur et al., 2014; Rilling and Young, 2014). We implanted tetrodes in the MeA of male mice (using 64 channels in four mice and 128 channels in five mice—a total of nine mice) and monitored their neural activity while the mice engaged in four typical social interactions, such as the social recognition of female faces, female anogenital areas, male faces and male anogenital areas (Figure 3A). A total of 495 well-isolated units were analyzed for their responsiveness during dynamic social interactions from these nine mice. Of those, 239 of them responded to these social-interaction events and they exhibited specific-to-general response patterns (Figure 3B). Interestingly, both hierarchical-clustering analysis and population *Z*-score analysis showed that these responsive cells followed the power-of-two-based permutation logic and had 15 permutated neural cliques to represent four distinct types of social information (Figure 3C). These results suggest that the MeA circuit also follows the specific-to-general permutation logic to register not only specific social recognition, but also to discover various combinatorial relationships of conspecific social interactions.

### The Power-of-Two-Based Logic for Processing Fearful Experiences

**Figure 4:**
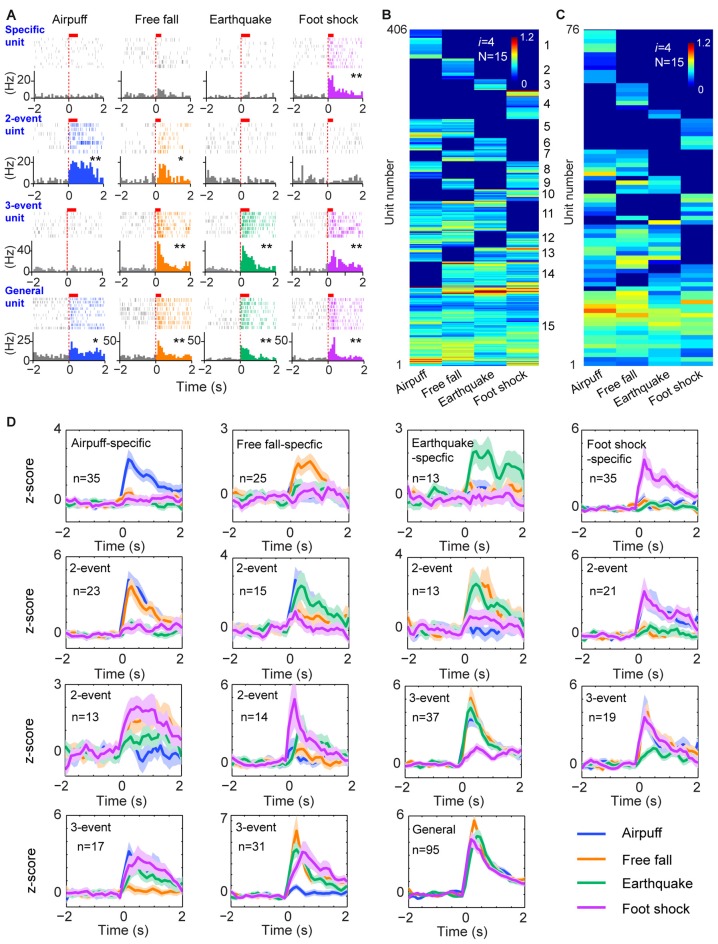
**The power-of-two-based logic in processing fearful experiences in the anterior cingulate cortex (ACC). (A)** Four distinct fearful experiences—airpuff, free-fall, earthquake and foot-shock—triggered robust firing increases in many ACC principal excitatory cells. Examples of specific-to-general responsive cells (list from the top to bottom rows, respectively). Red bars above the peri-event raster plots indicate the stimulus time-duration. *P < 0.05, **P < 0.01, Wilcoxon signed-rank test. **(B)** Hierarchical clustering plot shows the existence of 15 distinct principal neuron cliques in the ACC in processing four distinct fearful experiences. The plot showed 406 principal excitatory cells pooled from nine mice. **(C)** Hierarchical clustering plot of 15 specific-to-general ACC excitatory cell cliques from a single mouse data. Color scale bar indicates the scaled responsiveness. **(D)** *Z-score* plots of 15 distinct principal ACC neuron cliques from a single mouse (76 excitatory cells). Specific to general cliques are listed in rows from the top to bottom. The number of units belonging to each clique is indicated inside the plot. Colored lines indicate distinct fearful events.

To further test the generality of this logic in processing a wide range of cognitive information, we investigated how cell assemblies in the prefrontal cortex encode emotionally fearful experiences. We mimicked the accumulative experiences in real life by exposing the animals to four distinct fearful events (using air-puff blast, free-fall, earthquake-like shake and mild electrical foot-shock) known to trigger emotional responses (Liu et al., 2013, 2014). A total of 776 units were recorded using 128-channel tetrode arrays from the ACC, a region crucial for controlling fear behavior (Steenland et al., 2012) in freely behaving mice (*n* = 5 mice). Of them, 602 units met the criteria to be well-isolated. These units were then separated into putative excitatory units and fast-spiking interneurons (Supplementary Figures S2A,B). Stable units before, during and after fearful stimulation were selected for further analysis (Supplementary Figures S2C,D), and 520 putative excitatory units were identified. Many ACC excitatory cells (406 units) increased their firings to these fearful stimulations in a specific-to-general manner (Figure 4A). A total of 15 distinct principal cell cliques were identified based on hierarchical clustering analysis from the pooled datasets (Figure 4B). More importantly, single-mouse datasets also contained 15 distinct excitatory cell cliques (Figure 4C), suggesting that this power-of-two-based logic is also conserved in the individual ACC circuit. *Z-score* analysis of population datasets once again confirmed that these 15 cell cliques exhibited robust responsiveness (Figure 4D). Taken together, the above results show that the specific-to-general cell-assembly coding is based on the power-of-two-based permutation logic to extract every possible representational pattern across many cognitive modalities.

### The Power-of-Two-Based Logic is Repeatedly Utilized Along the Neural Pathways

Next, we examined the second prediction that the power-of-two-based permutation logic should be repeatedly utilized throughout various stages of network processing. Accordingly, we investigated cell-assembly patterns in three additional neural circuits known to be crucial for associative fear memories—namely, the infralimbic cortex (IL), RSC and CA1 region of the hippocampus (Kim and Jung, 2006; Do-Monte et al., 2015; Giustino and Maren, 2015).

**Figure 5:**
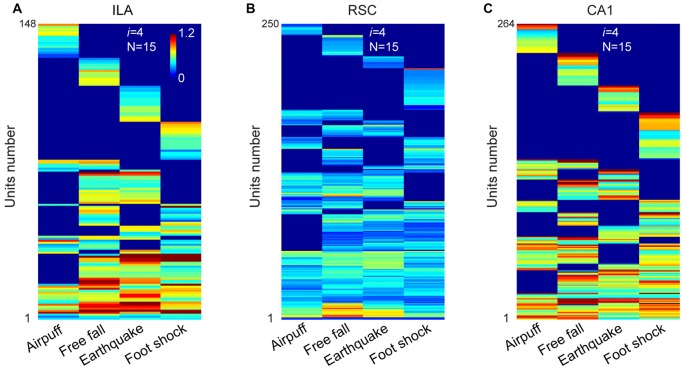
**The permutation logic is conserved in multiple cortical and hippocampal regions processing fearful experiences. (A)** Hierarchical clustering plot shows the existence of 15 distinct cell cliques in the ILA (148 out of 205 principal excitatory units responded to fearful stimuli from five mice). Color scale bar indicates the scaled responsiveness.** (B)** Hierarchical clustering plot shows the existence of 15 distinct cell cliques in the retrosplenial cortex (RSC). The datasets were pooled from nine mice (250 responsive excitatory cells out of a total of 346 units). **(C)** Hierarchical clustering plot shows the existence of 15 distinct CA1 pyramidal cell cliques. The datasets were pooled from five mice (264 responsive pyramidal out of 418 stable pyramidal cells). The *Y*-axis indicates the unit number.

A total of 423 units were recorded from the IL in freely behaving mice (*n* = 5 mice), and 296 units were judged as stable and well-isolated. Of them, 205 units were classified as putative excitatory units (Supplementary Figure S2B). Our large-scale recordings again detected 15 distinct neural cliques (from 148 responsive units) showing the specific-to-general coding patterns (Figure 5A and Supplementary Figure S3A).

Next, we investigated neural activation patterns in the RSC, a region implicated for long-term storage of fear-related memories (Cowansage et al., 2014; Kwapis et al., 2015). A total of 716 units were recorded from nine mice, and 545 were found to be isolated and stable. Out of these, 346 were identified as putative excitatory units (Supplementary Figure S2B). Our analyses revealed that these RSC excitatory units (250 responsive units) also exhibited specific-to-general, combinatorial response patterns (Figure 5B and Supplementary Figure S3B).

Finally, in the CA1, a total of 749 units were recorded from five mice and, of them, 529 were stable and well-isolated. There were 418 units classified as putative CA1 pyramidal cells (Supplementary Figure S2B). Our analyses of these pyramidal cells (264 responsive units) in pooled data also uncovered 15 specific-to-general response types (Figure 5C and Supplementary Figure S3C). Moreover, the existence of 15 permutated pyramidal cell cliques was also found in single-mouse data (Supplementary Figure S4).

Taken together, the results from four different regions (ACC, IL, RSC and CA1) revealed that the power-of-two-based permutation logic was, indeed, repeatedly employed across various network stages of the emotional memory circuits.

### A Simpler Logic in Dopaminergic Neurons

**Figure 6:**
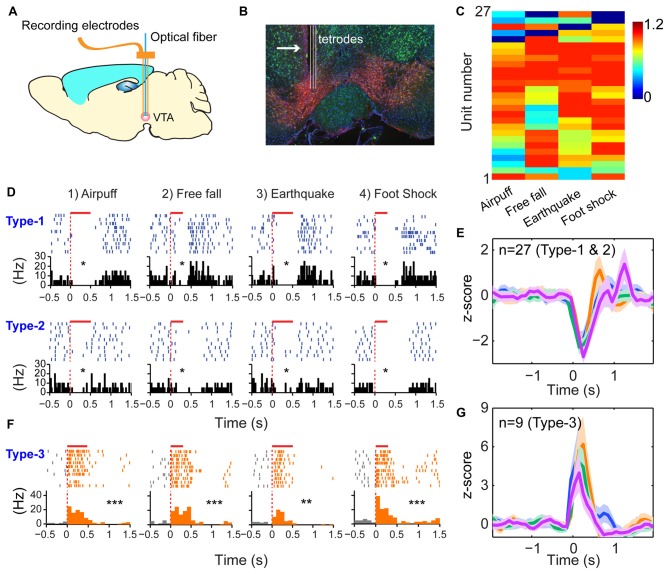
**Dopamine (DA) neurons did not use specific-to-general logic to process different types of fearful experiences. (A)** Tetrode recordings in the ventral tegmental area (VTA) in freely behaving mice. **(B)** Histological confirmation of the tetrode positions in the VTA. **(C)** Hierarchical clustering plot showed that DA neurons were broadly tuned to all four fearful events (81.5%), with 11.1% tuned to three events. Color scale bar indicates the scaled responsiveness. **(D)** Type-1 DA neuron suppressed its firing during fearful stimulation, followed by rebound excitation after the termination of the events, whereas Type-2 DA neuron suppressed its firing upon fearful stimulation but without rebound at the termination of the fearful events. The bar above each spike raster indicates the stimulus duration. *P < 0.05, Wilcoxon signed-rank test. **(E)** *Z-score* plot shows that DA Type-1 and -2 neurons (*n* = 27) exhibited a broadly tuned, significant suppression in their firings during fearful stimulation. **(F)** Type-2 DA neurons increased firing upon fearful stimuli and were broadly tuned to all four fearful stimuli. **(G)** *Z-score* plot shows that DA Type-3 neurons (*n* = 9) exhibited a significant increase of their firings upon all four types of fearful stimulation. **p < 0.01; ***p < 0.001.

The third prediction is that because the modulatory neurons—such as DA neurons in the VTA - have different functional purposes (i.e., providing motivational valence signals—such as wanting vs. not wanting, or rewarding vs. not rewarding, etc.). Accordingly, they may use a simpler logic. We recorded from the VTA in freely behaving mice (Figures 6A,B) while also subjecting them to the above four types of fearful stimulations. A total of 36 well-separated VTA putative DA neurons were identified from the VTA recordings (*n* = 10 mice) based on a combination of criteria—their waveforms (Supplementary Figure S5A) and decreased firing upon injection of D2 agonist apomorphine (Supplementary Figure S5B). In a subset of experiments, DA units were further confirmed by the optogenetic method (Supplementary Figures S5, S6). We noted that DA neurons can be classified into three major types. Type-1 and -2 DA neurons suppressed their firings during fearful stimulation, and these neuron DA subtypes (*n* = 27 units) were broadly tuned to these fearful stimulations (Figures 6C–E). Type-1 DA neurons (*n* = 24 units) exhibited significant rebound-excitation at the termination of fearful stimulation (Supplementary Figure S5E), whereas Type-2 DA neuron (*n* = 3 units) lacked such rebound-excitation (Supplementary Figure S5F).

In contrast to the suppressed firing upon fearful stimulation, Type-3 DA neurons (nine units recorded) increased firing to air-puff, free-fall, earthquake or foot-shock (Figure 6F), and they also lacked specificity (Figure 6G). Type-3 DA neurons were further confirmed by optogenetic and pharmacological methods (Supplementary Figures S6A–D). Taken together, despite their temporal dynamic differences, DA neurons lacked the power-of-two-based clique coding patterns and were broadly tuned to these distinct fearful stimuli.

### The Power-of-Two-Based Logic Remained Intact in the NMDA Receptor Knockout Mice

While Hebb postulated that cell assemblies are formed by learning, the *Theory of Connectivity* predicts that this power-of-two-based logic should be pre-configured by evolution and development. In other words, this logic should be independent of learning in adulthood. To examine these two competing hypotheses, we deleted the N-methyl-D-aspartate (NMDA) receptors in the forebrain excitatory neurons postnatally starting at 6-weeks’ old (Cui et al., 2004) and studied cell-assembly patterns (in BLA, RSC and ACC, respectively) of these adult conditional knockouts (KO) when they were 5–6 months old. This chronic deletion of the NMDA receptors throughout adulthood caused a random synaptic drift, which can destabilize synaptic patterns related to previously acquired memories, thereby reverting to the original naïve “unlearned” state (Cui et al., 2004, 2005).

**Figure 7:**
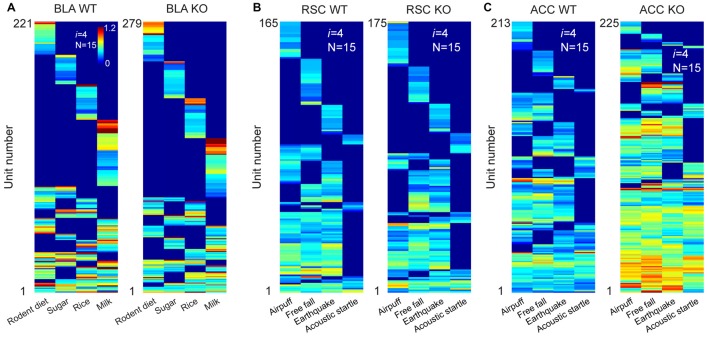
**The power-of-two-based logic was preserved in the N-methyl-D-aspartate (NMDA) receptor knockout (KO) mice. (A)** Hierarchical clustering plot shows the existence of 15 distinct pyramidal cell cliques in the BLA of the wild-type (WT) mice (221 cells) and KO mice (279 cells). Appetitive stimuli were labeled in the *X*-axis. **(B)** Hierarchical clustering plot shows the existence of 15 distinct excitatory cell cliques in the RSC of the WT mice (165 cells) and KO mice (175 cells). Four distinct fearful stimuli were labeled.** (C)** Hierarchical clustering plot shows the existence of 15 distinct excitatory cell cliques in the ACC of the WT mice (213 cells) and KO mice (225 cells) in response to four distinct fearful events. Color scale bars indicate the scaled responsiveness of these units.

We first recorded appetitive coding patterns from the BLA of the mutant mice as they were exposed to four distinct foods (mouse chew pellets, sugar pellets, rice and milk droplets). A total of 659 putative pyramidal units were identified from seven KO datasets. Of them, 279 cells responded to these four distinct foods. Fifteen distinct specific-to-general neural cliques were found among these pyramidal cells to represent these four food experiences in a surprisingly similar way to that of WT mice (Figure 7A).

We then investigated fear memory-coding patterns from the RSC of the KO mice (*n* = 6) and control mice (*n* = 6) while they were exposed to four distinct fearful stimuli (air-puff, acoustic startle, earthquake and free-fall). We obtained a total of 295 putative excitatory units from the RSC of the KO mice and 326 units from the control mice. Of them, 175 units out of 295 cells in the KO mice and 165 units out of 326 WT cells reacted to these fearful stimuli. Once again, we also identified 15 specific-to-general neural cliques in the RSC of both the KO mice and WT littermates (Figure 7B). These patterns were also very similar.

Finally, we recorded from the ACC of yet another set of the KO mice (*n* = 4) and control mice (*n* = 3) while exposing them to air-puff, acoustic startle, earthquake and free-fall. A total of 249 or 302 putative excitatory cells were identified from the KO and WT ACC region, respectively. 225 units out of those 249 cells in the KO mice and 213 units out of those 302 cells responded to these fearful events. There were 15 distinct specific-to-general cliques in both the KO datasets and their WT datasets (Figure 7C). Intriguingly, we noted that the ACC units in the KO mice exhibited greater firing increases in comparison to that of WT mice. Moreover, the general clique is much higher in proportion (40% in KO vs. 16% in WT), whereas the numbers of specific cells in the KO mice were greatly reduced (9%) in comparison to the 28% in the WT mice. This occurred while the basal firing rates in the BLA, RSC and ACC in the KO mice were comparable to those of the WT mice (data not shown). Taken together, these results show that the specific-to-general permutation logic remained intact after the NMDA receptors were deleted from the principal neurons in the forebrain throughout adulthood.

### Specific-to-General Cell Assemblies Deviated from the Random-Connectivity Model

Theoretically, in the early stage of evolution, a simple circuit with only several neurons could use the random-wiring strategy to generate specific-to-general combinatorial patterns. Also, in a given recurrent network with sufficient convergence and divergence, the random wiring-based mechanism may also be potentially employed to produce specific-to-general combinatorial connectivity. Thus, we asked whether specific-to-general clique ratios may match the random distribution. Because lack of the NMDA receptors causes a random drift in synaptic strength, we also wondered if deleting NMDA receptors in the adult brain would result in random-connectivity patterns.

**Figure 8:**
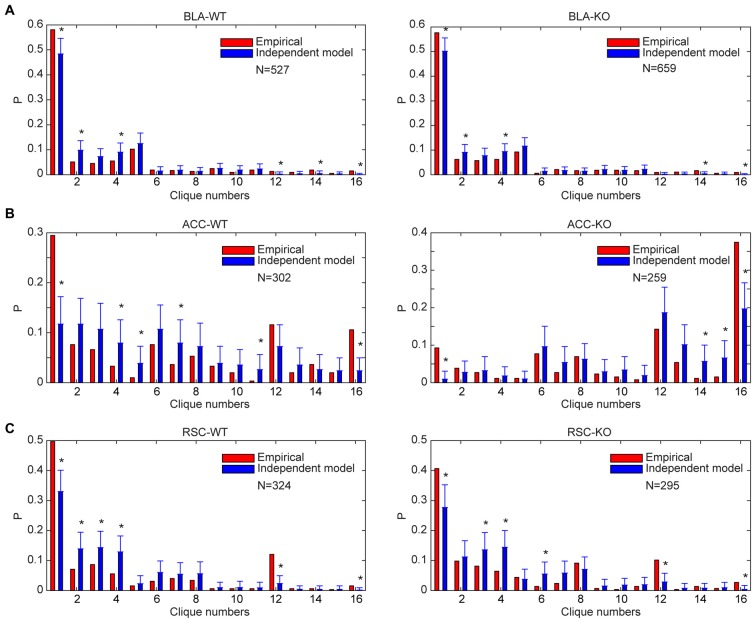
**Distribution of specific-to-general neural cliques in the BLA, ACC and RSC regions of the WT and KO mice deviated significantly from the random model. (A)** Distribution of response patterns in the BLA for WT mice and KO mice as fit by independent model. Distribution probability (*P*, *Y*-axis) in both WT and KO mice deviated significantly from the random model, *P < 0.05. **(B)** Distribution of response patterns in the ACC for WT mice and KO mice as fit by independent model. Distribution in both WT and KO mice deviated significantly from the random model. *P < 0.05. **(C)** Distribution of response patterns in the RSC for WT mice and KO mice as fit by independent model. Distribution in both WT and mutant mice deviated significantly from the random model. *P < 0.05. In *X*-axis, Clique #1 indicates non-responsive cells; Cliques #2–5 are one-event specific cliques; Cliques #6–11 are two-event cliques; Cliques #12–15 are three-event cells; and Clique #16 is general clique responded to all four stimuli.

Accordingly, we performed the independent random-connectivity model analysis on both the WT and mutant BLA, ACC and RSC datasets. We found that the experimentally observed specific-to-general patterns in the wild mice deviated significantly from the random model. Chronic deletion of the NMDA receptors throughout adulthood did not cause much of a drift from the nonrandom distribution patterns in the BLA, ACC and RSC to randomness (Figure 8); again, strongly indicating that the specific-to-general computational logic was constructed via the nonrandom strategy that is independent of learning in adulthood.

### Implementation of the Power-of-Two-Based Permutation Logic Within Cortical Layers

The fifth prediction made by the *Theory of Connectivity* is that the power-of-two-based computational logic should be implemented vertically in the cortex via differential distribution of specific-to-general cliques across laminar layers (termed as the cortical FCM; Tsien, 2015a; Li et al., 2016). While the primary sensory cortices (koniocortex) and primary motor cortex have the classic six layers (Harris and Shepherd, 2015), many associational cortices in the rodents or other animal species, including the ACC, PrL, RSC or piriform cortex only have a three-layered structure (namely—L1, L2/3 and L5/6; Hooks et al., 2011; Sugar et al., 2011; Ueta et al., 2013; Dembrow and Johnston, 2014; Fournier et al., 2015; Yamawaki et al., 2016). While L2/3 neurons project locally to L5/6, they are predominantly responsible for cortical-to-cortical communications. On the other hand, L5/6 neurons have recurrent local feedback to the superficial layers, and they project preferentially to subcortical systems. The *Theory of Connectivity* predicts that specific clique cells should be enriched in the superficial layers two and three (L2/3), whereas general clique cells should be concentrated in the deep layers five and six (L5/6; Tsien, 2015a; Li et al., 2016). Most importantly, the power-of-two-based permutation logic across laminar layers would ensure every possible combinatorial excitatory cell clique to be found within each cortical FCM.

**Figure 9:**
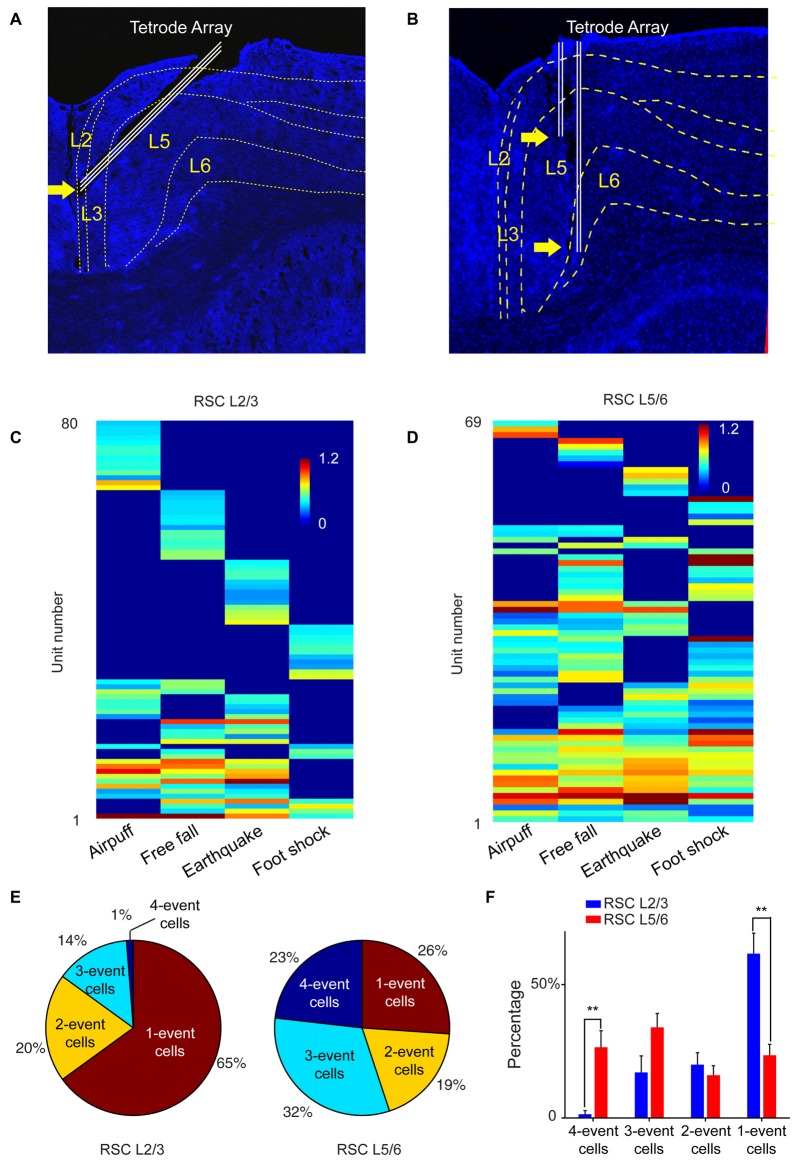
**The power-of-two-based logic computational logic is implemented through the cortical layers. (A)** Histological confirmation of the tetrode positions in L2/3 of the RSC of the mouse brain.** (B)** Histological confirmation of the tetrode positions in L5/6 of the RSC.** (C)** Hierarchical clustering plot revealed the highest proportion of cliques in L2/3 belongs to specific cliques, followed by two-event cliques. **(D)** Hierarchical clustering plot revealed that the general four-event clique and three-event cliques constituted a larger proportion. **(E)** Ratio of one-event, two-event, three-event and four-event excitatory cells between L2/3 vs. L5/6.** (F)** There are significant differences in the distributions of specific cells vs. the general cells between L2/3 and L5/6. Two asterisks denoted that the *p*-value is in the range of 0.01–0.001.

To test this prediction, we targeted our tetrode arrays specifically to the RSC L2/3 or L5/6, respectively (Figures 9A,B). We recorded from RSC L2/3 in nine mice while they were exposed to four types of fearful stimulations (namely, air-puff, drop, earthquake and foot-shock). Population analysis of 197 putative excitatory cells revealed that specific cells (responding to only one fearful event) constituted about 65% of all responsive cells. Only 1% of the responsive cells belonged to the general clique (four-event cells), and 14% of the responsive cells were three-event clique cells (Figures 9C,E).

We then recorded from L5/6 of the RSC in a different set of mice (*n* = 11) while the same four types of fearful events were delivered. Hierarchical clustering analysis of 168 putative excitatory cells showed that the percentage of cells belonging to one-event specific cells was significantly smaller (26%; Figure 9D). At the same time, the general four-event cells were expanded to 23% (Figures 9E,F). We found that three-event and four-event cells together constituted about 55% of the responsive population. These results demonstrated that the superficial layers preferentially contained many specific cliques, whereas the general cliques were greatly enriched in the deeper layers. Together, these vertically implemented computational motif (cortical FCM) followed the power-of-two-based permutation logic and covered all 15 specific-to-general coding patterns.

### The Power-of-Two-Based Permutation Logic is Evolutionarily Conserved

**Figure 10:**
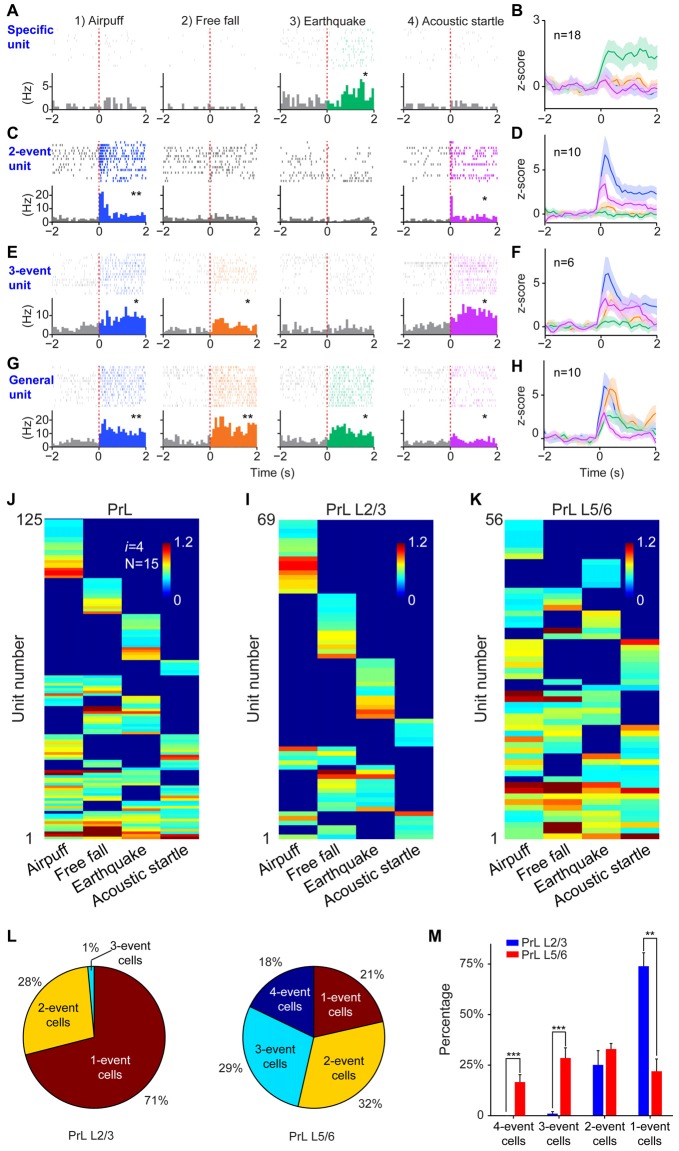
**The power-of-two-based computational logic was also observed in the prelimbic cortex (PrL) of the hamster brain. (A)** A specific cell responding specifically to earthquake. *P < 0.05, Wilcoxon signed-rank test. **(B)** The population response of this specific neural clique. **(C)** An example of two-event cell responding to two types of fearful events. *P < 0.05, **P < 0.01, Wilcoxon signed-rank test. **(D)** The population response of this two-event neural clique. **(E)** An example of three-event cell responding to three types of fearful events. *P < 0.05, Wilcoxon signed-rank test. **(F)** The population response of this three-event clique. **(G)** A general cell responding to all four types of fearful experiences as evidenced from the peri-event raster (upper subgraph) and peri-event histograms (lower subgraph). *P < 0.05, **P < 0.01, Wilcoxon signed-rank test. **(H)** The population response of the general neural clique responded to all four fearful events (*Z*-score plot). **(I)** Hierarchical clustering plot shows the existence of 15 distinct principal neuron cliques in the PrL. Unit number is labeled in the *Y*-axis. Color scale bars indicate the scaled responsiveness of these units. **(J)** Hierarchical clustering plot of L2/3 putative principal cells revealed the highest proportion of cliques belongs to specific cliques, followed by two-event cliques. **(K)** Hierarchical clustering plot of L5/6 principal cells revealed that the general clique and three-event cliques constituted a larger proportion. **(L)** Pie plots showed the ratio of one-event, two-event, three-event and four-event cells in L2/3 vs. L5/6. **(M)** There are significant differences in the distributions of specific cells vs. the three-event cells and general cells between L2/3 and L5/6. Two asterisks denoted that the *p-value* is in the range of 0.01–0.001, three asterisks denoted the *p-value* is < 0.001.

Finally, we tested the sixth prediction that the power-of-two-based computational logic should be evolutionarily conserved across animal species. We asked whether the PrL in Golden Syrian hamsters (Markham et al., 2012) would follow this logic in processing fearful experiences. We implanted 64-channel tetrode arrays bilaterally into the PrL region (L2/3 or L5/6) and recorded neural activity patterns while subjecting the hamsters to the same four types of fearful events that we used in mice (namely, air-puff, free-fall, earthquake and acoustic tone). A total of 336 units were recorded from 12 hamsters. Of these, 244 putative excitatory units were identified as stable and well-isolated (145 cells from L2/3, and 99 cells from L5/6). Many of them (125 units) responded to these fearful stimulations and also showed specific-to-general coding patterns (Figures 10A–H). Together, these putative principal cells in the hamster PrL cortex also followed the power-of-two-based permutation logic and formed all 15 distinct neural cliques to cover all possible coding patterns for these four fearful experiences (Figure 10I).

Further, we asked whether differential distributions of specific-to-general cliques observed in the mouse RSC would also be conserved in the hamster PrL. Thus, we analyzed these 125 responsive excitatory units according to their layer-specific distributions. Indeed, we found that specific clique cells were again overwhelmingly concentrated in the L2/3 of the hamster PrL cortex (Figure 10J), whereas the general responsive cells and most of the three-event cells concentrated in the L5/6 (Figure 10K). Quantitative analysis revealed that 71% of the all responsive cells in L2/3 belonged to specific cliques (Figure 10L). Only 1% of these responsive cells reacted to three-events, and there was no four-event unit. On the contrary, a greater percentage of the excitatory units in L5/6 belonged to the general clique (18%) and three-event cells (29%; Figure 10L). Statistically, there were significant differences in the distributions of one-event units vs. three-or four-event cliques between L2/3 and L5/6 in the hamster PrL cortex (Figure 10M). These results demonstrated that the power-of-two-based computational logic is evolutionarily conserved in the hamster brain, and so is its cortical architectural implementation of specific-to-general cell cliques.

### Randomness in Superficial Layers and Nonrandomness in Deep Layers

L2/3 neurons are known to be responsible for cortical-to-cortical communications, whereas L5/6 neurons project predominately to subcortical systems. As such, these cortical cells are clearly organized for different functional purposes. Thus, we took advantage of the layer-specific identification of all 15 neural cliques and investigated further whether different cortical layers employ a different connectivity strategy in implementing the permutation-based computational logic. Specifically, we asked whether distinct specific-to-general clique distributions in L2/3 vs. L5/6 might indicate their underlying organizing principles.

**Figure 11:**
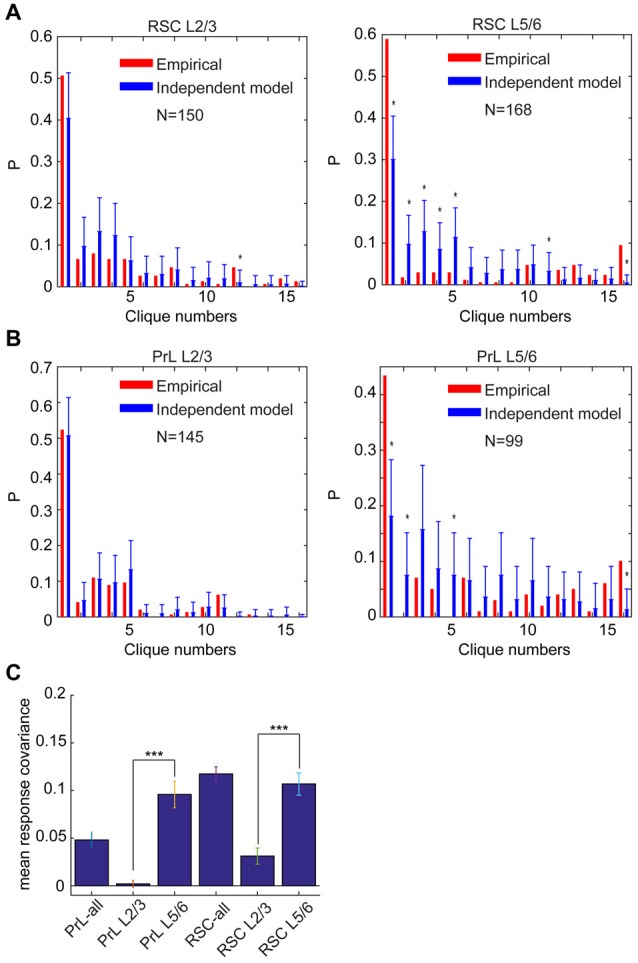
**Specific-to-general cortical clique distributions exhibited randomness in L2/3 but nonrandomness in L5/6. (A)** Distribution of response patterns in the mouse RSC for layers 2/3 and layers 5/6 as fit by independent model. Distribution in L2/3 was closer to the random model, whereas L5/6 deviated significantly from the random model. *P < 0.05. **(B)** Distribution of response patterns in the hamster PrL for layers 2/3 and layers 5/6 as fit by independent model. Distribution in L2/3 was closer to the random model, whereas L5/6 deviated significantly from the random model. *P < 0.05. In *X*-axis, Clique #1 is non-responsive cells; Cliques #2–5 are one-event specific cliques; Cliques #6–11 are two-event cliques; Cliques #12–15 are three-event cells; and Clique #16 is general clique responded to all four stimuli. **(C)** The covariance was significantly higher in L5/6 than L2/3 in both the PrL of the hamsters and RSC of the mice. ***P < 0.001.

Accordingly, we performed the independent-connectivity model analysis on the layer-specific RSC datasets. Surprisingly, our analyses revealed that L2/3 cliques in the mouse RSC resembled to random distribution (Figure 11A). In contrast, neural cliques in L5/6 showed high nonrandom probability distribution patterns (Figure 11A).

Most interestingly, the similar randomness in L2/3 and highly nonrandomness in L5/6 were also observed in the PrL of hamsters (Figure 11B). This stark difference between L2/3 and L5/6 patterns was also clearly evident from covariance analyses of L2/3 vs. L5/6 clique distribution-probability (Figure 11C). Given that L2/3 is the major gateway for inter-cortical communication, this finding supports the notion that randomness can be ideal to discover novel cross-modality association, enabling pattern-discrimination and pattern categorization, whereas nonrandomness in L5/6 cliques, which mostly project to subcortical structures and contain more general cliques for categorized generalization, is ideal to ensure robust execution of evolutionarily selected, adaptive behaviors.

## Discussion

In the present study, we systematically tested the six predictions made by the *Theory of Connectivity*. We show that this power-of-two-based permutation logic operated in seven different brain regions and in two animal species during processing appetitive, emotional and social experiences. These findings are quite revealing to us for the following reasons.

First, this mathematical principle is strikingly simple, yet it is this power-of-two-based permutation logic that can best deal with uncertainties and endless possibilities by generating every connectivity pattern. In other words, the resulting FCM—in the form of specific-to-general cell assemblies—covers not only specific patterns but also every possible combination of these patterns. Moreover, the assemblies organized by this logic can intrinsically give rise to pattern-discrimination, pattern-categorization and pattern-generalization as animals’ experiences accumulate over time, forming the microcircuit basis for specific memories and actions, categorical knowledge and skills, general concepts and motor commands.

**Figure 12:**
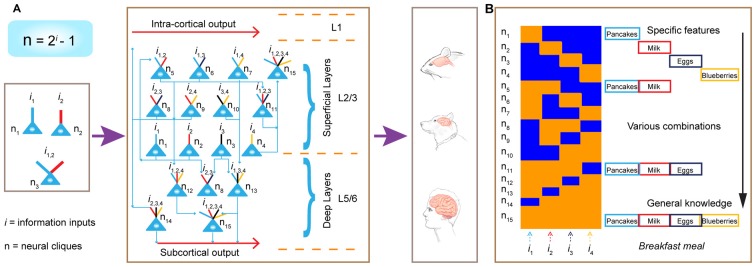
**Power-of-two-based permutation logic governs brain evolution and cortical expansion. (A)** The logic is applicable from the simplest circuits (1 st left subpanel) during the early evolution of the complex cortex in humans. Implementation of the power-of-two-based computational logic in the cortex via differentially hosting various cliques across laminar layers. In the classic 6-layered cortex, the layer 4 hosts most of the specific cliques. In the 3-layered cortex (illustrated here), there is no layer 4. As such, the cortex is divided into the L1, the superficial layers (L2/3) which preferentially host low-level combinatorial cells, and deep layers (L5/6) which host more high-level combinatorial cells. The implementation of this power-of-two-based logic can be repeatedly utilized as cortical expansion occurred over evolution. **(B)** Illustration of the emergence of specific features, sub-combinatorial features, as well as the abstract concept of “meal” from specific-to-general cell assemblies in the appetitive circuit. This ensemble activation pattern allows pattern-separation, pattern-categorization and pattern-generalization of various food experiences at the cognitive level.

Second, this power-of-two-based permutation logic fulfills the critical requirement as the unifying design principle, because it can preside brain computation across the entire evolutionary spectrum—ranging from the simplest neural networks to the most complex (Figure 12). This universality is experimentally demonstrated by the observation of the same logic in seven different neural circuits, including the nonrecurrent networks such as the CA1 region and BLA, as well as the recurrent networks such as the cortex.

Third, this logic is also capable of describing the general computational purpose of the cortex regardless of the evolutionary variations in cortical structures and modality functions. The fact that all four cortices (the ACC, PrL, ILA and RSC) contained 15 specific-to-general cell assemblies is consistent with the notion that this power-of-two-based permutation logic serves as the general computational algorithm of the cortex.

### Brain Computation and Its Evolutionary Logic

Over evolution, different animals have developed a variety of drastically different sensory apparatus (from typical sensory organs to exotic ones—such as electroreception in electric eels, magnetoception in homing pigeons, sonar in dolphins and infrared visions in bedbugs; Teeling et al., 2000; Clarke et al., 2013). As such, different animals construct very different perceptions of the world in their brains. Likewise, behavioral skills have also evolved differently—from swimming, flying and walking, to playing basketball or managing business and governments. As an individual, the animal would typically encounter numerous events, objects, foods and countless social interactions in a lifetime. Therefore, from an evolutionary perspective, the central mission of the brain is to discover various meaningful patterns from natural and social environments and subsequently convert them into specific memories, categorical knowledge and flexible behaviors. Such cognitions and behaviors require the brain’s neural circuits to be capable of pattern separation, categorization, and generalization. The power-of-two-based permutation logic provides the cell-assembly level solution to this problem. It affords the brain the unique ability to cope with a complex, ever-changing world full of uncertainties and infinite possibilities of which current computers are incapable (von Neumann, 1958; Hawkins and Blakeslee, 2007; Legg and Hutter, 2007; Brenner and Sejnowski, 2011). Evolution can then repeatedly utilize this mathematical principle to construct various brains from the simplest organisms to the most complex mammals.

In the early stages of evolution when animals began to appear 500–600 million years ago, the random connectivity strategy may be initially used to execute this power-of-two permutation-based mathematical logic by constructing a simple circuitry node for rudimentary pattern-separation and pattern-generalization (Tsien, 2015a,b). For example, in a three-neuron circuit, one neuron encoded foods, another neuron encoded mates, the third neuron (which received convergent inputs from the food neuron and the mate neuron) encoded combined information and generated motor intents and commands such as “attractive and approaching” (Figure 12A, left panel). This similar arrangement can be applied to an aversive neural node to generate escape behavior in order to avoid obstacles and dangers (e.g., hot rocks or predators). Through evolution as more neurons became available, the brain evolved with a greater capacity to expand the “power-of-two”-permutation-based permutation wiring and consequently, to extract more relational patterns (*i* gets larger as *N* is bigger based on the equation of *N* = 2^*i*^ − 1), thereby leading to higher abstraction of categorical knowledge and more intelligent behaviors. Over time, when the random connectivity strategy may no longer be sufficient and efficient to ensure the desired outcome, evolution exerts its selection force to develop nonrandom organization to execute this power-of-two-based permutation logic to efficiently deal with environments in which animals lived. This is supported by our findings that specific-to-general cell assembly patterns in the CA1, BLA and cortex (combined from L2–6) all differed significantly from the independent random-connectivity model.

Interestingly, within the cortical architecture, our analysis suggests that evolution employed both random and nonrandom connectivity strategies to construct its laminar layers—namely, randomness for L2/3 organization vs. nonrandomness for L5/6 organization. Once this cortical FCM was in place (Figure 12A, middle panel), evolution can seamlessly implement this power-of-two-based permutation logic via expanding its surface size horizontally (Figure 12A, left panel). This is supported by the examination of 30 different animal species showing that cortical surface area varied by a factor larger than 10,000, whereas the thickness of the cortical layers varied only by a factor of 10 (Hofman, 1989). The horizontal expansion via repeated utilization of these cortical FCMs can explain how the more complex brains emerge, equiped with greater conceptual knowledge and intelligence (Hofman, 2014; Dicke and Roth, 2016).

At the cognitive level, the specific-to-general cell-assembly logic offers the network-level explanation as to how the brain can encode not only specific memories but also categorical knowledge (Lin et al., 2006b; Tsien, 2007; Tsien et al., 2013). As evident from identification of specific-to-general pyramidal cells in the BLA responding to food experiences, one can envision that such a cell-assembly logic can allow the appetitive circuits to represent not only specific food experiences but also various combinations of different types of foods, giving rise to the general concept of “meals” (Figure 12B). In the past, specific memory and conceptual knowledge have been often studied as separate problems with the belief that they may engage different cellular mechanisms. To the contrary, our results suggest that specific memory and generalized knowledge are generated via a coherent cell-assembly logic and should emerge simultaneously. This suggests that pattern-separation and pattern-generalization can be widely studied in a variety of circuits far beyond the hippocampus as David Marr had originally envisioned (Marr, 1971; Leutgeb et al., 2007; McHugh et al., 2007; Clelland et al., 2009; Rolls, 2013; Xu and Südhof, 2013). It is noteworthy that this logic can also be applied to motor circuits to generate specific motor action and categories of motor behavior, but in a reversed general-to-specific manner (Li et al., 2016).

### What Do Randomness in L2/3 and Nonrandomness in L5/6 Mean for Sparse Coding, Memory Consolidation and Adaptive Behaviors?

The power-of-two permutation-based computational logic can be executed in the cortex whether it has three or six layers (Barbas, 2015; Fournier et al., 2015), in the service of the fundamental cortical “processing unit” that has been long sought-after (Van Hemmen and Sejnowski, 2005; Rabinovich et al., 2008; Hill et al., 2012; Marcus et al., 2014; Adolphs, 2015; Lisman, 2015). In both the mouse RSC and hamster PrL, we showed that specific cells were concentrated in the superficial layers (L2/3), whereas general cells were enriched in the deep layers (L5/6). This is consistent with literature on primary sensory cortices (i.e., L5/6 cells in the auditory cortex of anesthetized rats had broader frequency-tuned responses than L2/3 cells; Sakata and Harris, 2009; Harris and Shepherd, 2015). By taking advantage of our successful identification of all 15 specific-to-general neural cliques, we compared their distribution-probability patterns with that of an independent random-connectivity model. Surprisingly, we discovered that L2/3 specific-to-general cliques in both mice and hamsters conformed to the randomly produced patterns, whereas neural cliques in L5/6 showed high nonrandomness. This finding can account for the discrepancy in the literature whether cortical patterns are random or nonrandom (Szentágothai, 1989; Sjöström et al., 2001; Holmgren et al., 2003; Song et al., 2005; Carlo and Stevens, 2013). L2/3 randomness and L5/6 nonrandomness are highly informative to us for several reasons.

First, L2/3 neurons are the major sources for inter-cortical communications, whereas L5/6 neurons mainly project to subcortical systems (Hooks et al., 2011; Sugar et al., 2011; Ueta et al., 2013; Dembrow and Johnston, 2014; Barbas, 2015; Fournier et al., 2015; Harris and Shepherd, 2015; Yamawaki et al., 2016). This suggests that superficial layers and deep layers have completely different missions. Our observed random strategy in generating L2/3 specific-to-general cliques vs. nonrandom strategy in generating L5/6 specific-to-general cliques makes good sense from an evolutionary and a cognitive perspective. As information from different sensory modality needs to be integrated and extracted for higher cognitions, the randomness in constructing L2/3 neural cliques is ideal to maximize the capacity in discovering various possible novel combinations across a wide range of sensory cortices. The power-of-two-based permutation logic mathematically guarantees every possibility would be covered at the structural and computational levels. The observation that specific cells and low-combinatorial cells are concentrated in the L2/3 also makes it suitable for pattern-separation and pattern-categorization of abstract concepts and specific knowledge. By communicating with sensory cortices, L2/3 neurons in the associational cortex and prefrontal cortex generate distinct assembly patterns for specific memories and facts.

Second, this local L2/3 randomness of synaptic connectivity can be useful for not only rewiring synaptic connections by experience-dependent plasticity during development, but also during rehabilitation (Sur et al., 1988; Recanzone et al., 1993; Rothschild and Mizrahi, 2015; Hua et al., 2016). Independent of the intrinsic changes of molecular and cellular composition of neurons and circuits (Monyer et al., 1994; Sheng et al., 1994), this L2/3 randomness principle provides another explanation as to why such a re-wiring process is difficult and slow.

Third, it also provides an experimental evidence for why cross-modality learning of knowledge can be slower than the formation of simple memory (Lisman and Morris, 2001). This is because the L2/3 randomness deems their synaptic efficacy in across-modality connectivity to be much less robust, leading to possible sparse code. Such coding strategy is useful for coding specific percepts and concepts (Song et al., 2005; de Kock et al., 2007; Lin et al., 2007; Hromádka et al., 2008; Waydo and Koch, 2008; Bowers, 2009). Yet, the disadvantage of this randomness-based sparse code is that post-learning spontaneous reverberation may be too weak to occur on its own. But it can be resolved via the offline trainings and coordination from the hippocampal system during memory consolidation (Squire, 1992; McClelland et al., 1995; Bontempi et al., 1999; Kudrimoti et al., 1999; Zola and Squire, 2000; Sara and Hars, 2006; Dudai et al., 2015). Therefore, the random strategy in organzing specific-to-general cell assemblies in the L2/3 can nicely explain why the hippocampal system (offline reverberation) is critical for memory consolidation and long-term cortical storage (Skaggs and McNaughton, 1996; Foster and Wilson, 2006; Moser et al., 2005; Takehara-Nishiuchi and McNaughton, 2008; Chen et al., 2009; Zhang et al., 2013). This coordinated cortical-hippocampal consolidation have been shown to require the NMDA receptor reactivation-mediated synaptic reentry-reinforcement (SRR) mechanism (Shimizu et al., 2000; Santini et al., 2001; Wittenberg and Tsien, 2002; Cui et al., 2004, 2005; Wu et al., 2007; Tsien et al., 2013), a property that can be examined in finer details within the context of this power-of-two-based logic.

In comparison, nonrandomness in L5/6 neural cliques is ideal for pattern-categorization and pattern-generalization to generate categorical behaviors, general commands and overall cognitive states. Their heavy projections to subcortical sites such as the amygdala, midbrain dopaminergic circuits, and striatum exert feedback controls of general motivational, emotional and conscious states, as well as behavioral outputs that were built and selected by evolution. In this sense, such states are relatively low-dimensional, but useful to ensure evolutionarily proven responses. This is in contrast with much higher dimensionalities that random L2/3 cliques use in dealing with almost endless possibilities that specific knowledge structure may face.

### The Power-of-Two-Based Permutation Logic Helps Explain Many Results in the Literature

This permutation logic-based coverage of all possible connectivity-patterns can mechanistically account for why researchers reported all sorts of interesting cells in the brain that corresponded to some kind of specific stimulus or multiple stimuli (e.g., syringes, peanuts, faces, hands, the actress Halle Berry, or a nest) or a category of items (e.g., dogs vs. cats, or people vs. other objects; Rolls et al., 1979; Logothetis and Sheinberg, 1996; Fried et al., 1997; Freedman et al., 2003; Hampson et al., 2004; Gross, 2005; Quiroga et al., 2008; Bowers, 2009; Tsao, 2014). Although combining simple stimulus-features from sensory organs for higher cognition were often postulated and reported (Buck and Axel, 1991; Yeshurun and Sobel, 2010; Fu et al., 2015), findings of some cells in a given site which responded to multiple stimuli (as the literature intermittently described in bits and pieces) have reinforced the popular but undue impression that somehow convergence and combination occurred but in a stochastic and random fashion. Obviously, such sporadic combinations can easily take place in various ways without conforming to the power-of-two based permutation logic. As a result, the stochastic convergence (in the absence of the systematic permutation logic) became a trivial mechanism which failed to be intuitively conceptualized into a general organizing principle under which orderly abstraction and intelligent cognition would emerge from the brain’s networks.

There is general agreement that information processing in the brain is hierarchical—that is, simple features in the primary sensory cortex are somehow transformed into complex features in the next-stage cortex, and so on and so forth. The demonstration of the power-of-two-based permutation rule in producing comprehensive cell cliques within each microcircuitry motif, arranged in a specific-to-general manner, provides the unifying microcircuit-level framework for achieving this hierarchical construction (Tsien, 2015a). This logic contrasts with many conventional models in which “simple feature-to-complex feature” conversion emerges from one region to the next in a two- or multi-staged sequential manner. Further investigation of this power-of-two-based permutation logic can shed crucial insights into how hierarchical representation is achieved in real-time along and among various neural pathways.

This logic can also explain well why initial orderly architectures in the primary sensory cortices, which often have structural motifs (i.e., mini-columns or patches dedicated to processing specific sensory inputs), (Mountcastle, 1957; Hubel and Wiesel, 1962; Vassar et al., 1994; Erzurumlu et al., 2010) have been discarded as modality-specific, early-stage sensory information is transmitted deeper into the association and decision-making circuits (Heisenberg, 2003; Rakic, 2008; Sosulski et al., 2011; Bergan et al., 2014; Rothschild and Mizrahi, 2015). This can be explained by the power-of-two-based cell-assembly logic aimed at discovering and maximizing various novel cross-modality patterns for specification as well as generalization. Such cell-assembly level organization does not necessarily need to occur in the form of histological “patches or columns.” In fact, the randomness, as discovered in the superficial layers for the execution of the power-of-two-based permutation wiring logic, is preferred.

While it is nice for the power-of-two-based permutation logic to account for much of the evidence seen in the literature, such extrapolation should be treated with great caution. For example, some of the studies contained a mixture of single-units and multi-units due to the insufficient spike-sorting resolution of single electrodes. Moreover, principal excitatory cells and interneurons usually were not separately characterized. Because interneurons tend to exhibit broad tuning properties, such inhibitory units could be misquoted as literature evidence for general or sub-general excitatory cell cliques. Another caveat is that repeated presentations of the same stimuli over a period of days, weeks, and even months may result in learning-induced association, habituation, or sensitization, thus altering their original response properties. Similarly in human studies, it has been reported that a hippocampal cell was activated by pictures of Jennifer Aniston and Lisa Kudrow—both actresses in the TV series Friends (Quiroga et al., 2008). It remains to be deteremined whether such a coding property reflects the associative learning after repeated experiences (such as watching the TV series for many weeks or months) or the stochastic input convergence that is independent of learning. In addition, many previously reported data were not collected from the same recording site(s) or subregion. Thus, gross extrapolation by taking one example from one region in an animal and linking it with another unit recorded from another site(s) in another animal species can be problematic. In light of our observed permutation logic, it may be beneficial to re-examine these fascinating issues in the future with large-scale recordings and imaging techniques.

### Power-of-Two-Based Logic and its Constraints on Biological Brains

While the power-of-two-based permutation logic is based on the mathematical equation of *N* = 2^i^−1, it imposes biological boundary on the computational limitation of circuits. For example, due to exponential growth in input numbers *i*, the cost (in terms of cell resources) can quickly become prohibitive. For instance, in order to cover all possible patterns for processing 40 distinct perceptual inputs, a single FCM would require over a trillion neurons (10^12^). Even neurons in the human brain (8.6 × 10^10^ neurons) would be inadequate to afford this exponential coverage if this power-of-two-based logic were to be rigidly applied. The best and necessary solution is to employ modular approaches, or a divide-and-conquer strategy, to segregate or stream information inputs through distinct sensory domains or pathways.

For example, if a central node in a small neural circuit needs to cover all possible connectivity wiring patterns to represent eight distinct types of inputs or information, a total of 255 principal projection neurons would be required (*N* = 2^8^ −1 = 255) for this node. However, when a sub-modular approach is employed (e.g., dividing into a set of four inputs per subnode), the same 255 principal neuron sets can increase its processing capacity by a factor of 17 times (255 total cells/15 cells per sub-node = 17). Similarly, if a subnode or FCM was structured to process only three distinct pieces of information (*N* = 2^3^ −1 = 7), 255 neurons can be used to construct 36 assemblies for processing 108 distinct information inputs. Through evolution, *i* number should have been selected and confined by the complexity of given environmental demands in which organisms have lived for generations. This means that evolution has been forced by this mathematical cost-and-benefit analysis to use neuron resources efficiently and wisely, as evident from the evolutionarily conserved specific sensory pathways and cortical modalities. This boundary condition can be tested in simple organisms such as drosophila larvae or *C. elegans* as well as classic mammalian models such as mice and rats by increasing numbers of inputs. Perhaps it should also be explored and expanded in computational modeling, neuromorphic chips and artificial intelligence systems.

### Neuromodulatory Neurons Such as DA Cells Use a Simpler Logic

Because the overall functional purpose of modulatory neurons is to provide motivational and attentional signals (i.e., to define positive or negative valence, or whether the brain should pay attention or not, or whether an animal is hungry or not, etc.), the *Theory of Connectivity* posited that neuromodulatory circuits should use a different, but more likely, simpler logic. This was indeed found in the dopaminergic circuit. While literature described that VTA DA neurons responded to the air-puff (to monkey’s eyelids) or to a tail-pinch or foot-shock in anesthetized rats (Brischoux et al., 2009; Matsumoto and Hikosaka, 2009; McCutcheon et al., 2012; Fiorillo et al., 2013), tuning properties over various types of fearful stimuli by the same DA neurons (whether in anesthetized or freely behaving states) were rarely examined. Here, we show that despite diverse dopaminergic neuron subtypes, they showed broad responsiveness to all four different fearful stimuli. These subtype DA neurons showed distinct temporal dynamics, with either suppressed firing or increased firings during aversive stimulation. However, they did not use the specific-to-general coding logic. Extending the similar multi-categorical investigations to other modulatory neuron types (such as serotonin, adrenergic or cholinergic neurons) will be necessary and informative.

### The Power-of-Two-Based Cell-Assembly Logic Does not Require Adult NMDA Receptors

One of the key questions is whether this power-of-two-based, cell-assembly logic should be formed by learning in adulthood or pre-configured by development, or both. We employed two approaches to examine the role of learning in our current experiments: one was to take steps to minimize learning in the present experiments, and another was to delete the NMDA receptors in the forebrain right after postnatal development.

In our experiments, various specific stimuli (such as foot-shock, drop, earthquake, air-puff, rice, etc.) were given for the first time to the recorded mice whenever possible (the exceptions are rodent diet and pre-weaned social interactions with their littermates). Our datasets were all collected in the very first set of trials (limited to seven trials per stimulus type without any former training). This ensured the patterns were not a result of repeated conditioning.

We then used forebrain NMDA receptor KO mice to assess whether learning is necessary to the observed coding logic. It is known that synaptic proteins are metabolically turned over within hours, days or week(s). This means that learning-induced synaptic connectivity should drift significantly over time (Shimizu et al., 2000; Wang et al., 2006; Choquet and Triller, 2013). We have shown that the NMDA receptor-based SRR is crucial for maintaining the stability of synaptic connections established by prior learning (Wittenberg and Tsien, 2002; Wittenberg et al., 2002). For example, inducible KO of the NMDA receptor in the forebrain principal neurons for one month (but not for one week) results in the abolishment of remote fear memories due to random drift in the synaptic connectivity established by previous learning (Cui et al., 2004, 2005; Wang et al., 2006). We reasoned that if specific-to-general coding logic was based on a random connectivity mechanism during development, one should expect that deleting the NMDA receptor throughout adulthood would eventually lead synaptic connectivity to drift all the way back to the initial randomness.

In the present study, the NMDA receptors in the forebrain were deleted, starting at the sixth postnatal week and throughout adulthood, which is known to produce profound deficits in appetitive, social and fear memories (Cui et al., 2004, 2005; Jacobs and Tsien, 2016). We previously also showed that loss of the NMDA receptors impaired the formation and reverberations of associative real-time fear memory traces in the hippocampal CA1 region (Zhang et al., 2013). Here, we recorded from three structures—namely, the BLA, RSC and ACC. Interestingly, we found that deleting the NMDA receptor had minimal effect on the specific-to-general connectivity logic in all three circuits, suggesting that the power-of-two-based logic is not dependent on learning in adulthood. This finding is in line with the emerging evidence that specific input patterns in the cortex were largely independent of NMDA receptor function (DeNardo et al., 2015). It also fits with the reports showing pre-organized spontaneous firing patterns or sequences in the visual cortex, hippocampus, and entorhinal cortex (Tsodyks et al., 1999; MacLean et al., 2005; Dragoi and Tonegawa, 2013; Mizuseki and Buzsáki, 2013). It will be of great interest to examine how neural ontogeny and circuit development lead to such a remarkably deterministic blueprint (Gao et al., 2014; Wilber et al., 2014).

### Further Testing and Additional Validation

It is widely believed that functional selectivity reflects the underlying structural connectivity. Although the specific-to-general coding patterns suggest their underlying wiring logic, the direct evidence for such wiring patterns awaits future demonstration. Perhaps it will be best approached by studying in simpler organisms (drosophila larvae) in which structural connectivity and functional imaging can be better analyzed (the Keyon cells in the mushroom body; Chiang et al., 2011; Wang et al., 2013). Moreover, our current cortical layer-specific recordings were limited to three-layered cortices, further testing in the koniocortex and motor cortex, which have six-layered cortices, will be necessary. It will also be crucial to examine whether and how this logic operates in monkeys and humans. In addition, we have previously shown that some of CA1 neurons which responded to drop events did not differentiate the environmental contexts, yet, some of those drop-sensitive units were also sensitive to specific context (Lin et al., 2005). For instance, some units responded only to drop inside Elevator-A, whereas other units responded only to Elevator-B, suggesting that those CA1 cells encoded for both “what and where” information (Lin et al., 2005, 2006b). Thus, it will be of considerable interest to examine how environmental and emotional contexts (including habituation vs. novelty) might affect cell-assembly response patterns in neural circuits.

Finally, while our present study has focused on principal projection neurons, it is clear that their dynamic patterns are critically modulated by local inhibitory interneurons (Freund and Buzsáki, 1996; Klausberger and Somogyi, 2008; Kätzel et al., 2011). Cre-lox neurogenetics (Tsien et al., 1996a; Tsien, 2016) combined with chemical-genetic and/or optogenetic manipulation (Lim et al., 2013) will be highly useful to delineate how various interneuron types compute information and modulate circuitry dynamics (Kvitsiani et al., 2013; Dembrow and Johnston, 2014; Kim et al., 2016). Collectively, such efforts will likely lead to a better understanding of how the brain’s logic is organized in specific circuits and how various other rules and properties might be integrated (McClelland et al., 1995; Klausberger and Somogyi, 2008; Perin et al., 2011; Shanahan, 2012; Stevens, 2012; Miller, 2016).

In summary, we present a series of experimental evidence for the *Theory of Connectivity* that a simple mathematical logic underlies brain computation. The power-of-two permutation-based logic governs specific-to-general cell-assembly coding patterns that are capable of pattern discrimination, categorization and generalization, giving rise to specific perception and memories as well as generalized knowledge and adaptive behaviors. Further testing of this power-of-two-based logic in additional neural circuits and animal species will be highly desirable, and exploring its applications in general artificial intelligence systems can also be informative.

## Author Contributions

JZT conceived the project and then designed the experiments with KX, HK, and JL. The research was performed as follows: KX generated the datasets from the ACC and MeA in mice and PrL from hamsters; GEF generated the datasets from the RSC in both WT (with HK) and KO mice; JL generated the datasets from the BLA in WT and KO mice; SJ and HK for the IL datasets; HK and JL for the CA1 datasets; JCL for the DA neuron datasets. CL, ML, HK and SS for data analyses together with KX, GEF, JL, JCL, TL and JZT. JZT wrote the article with input from all the other authors.

## Conflict of Interest Statement

The authors declare that the research was conducted in the absence of any commercial or financial relationships that could be construed as a potential conflict of interest.

## Footnotes

^1^http://redishlab.neuroscience.umn.edu/MClust/MClust.html

^2^http://www.plexon.com
