# Supplementary material for: Brain Computation Is Organized via Power-of-Two-Based Permutation Logic
Source: Front Syst Neurosci. 2016 Nov 15;10:95. doi: 10.3389/fnsys.2016.00095 (PMC5108790; doi:10.3389/fnsys.2016.00095)
Supplement: Supplementary file 1 [file Presentation_1.pdf]

Supplementary Figures for Xie et al., Frontiers in Systems Neuroscience, 2016

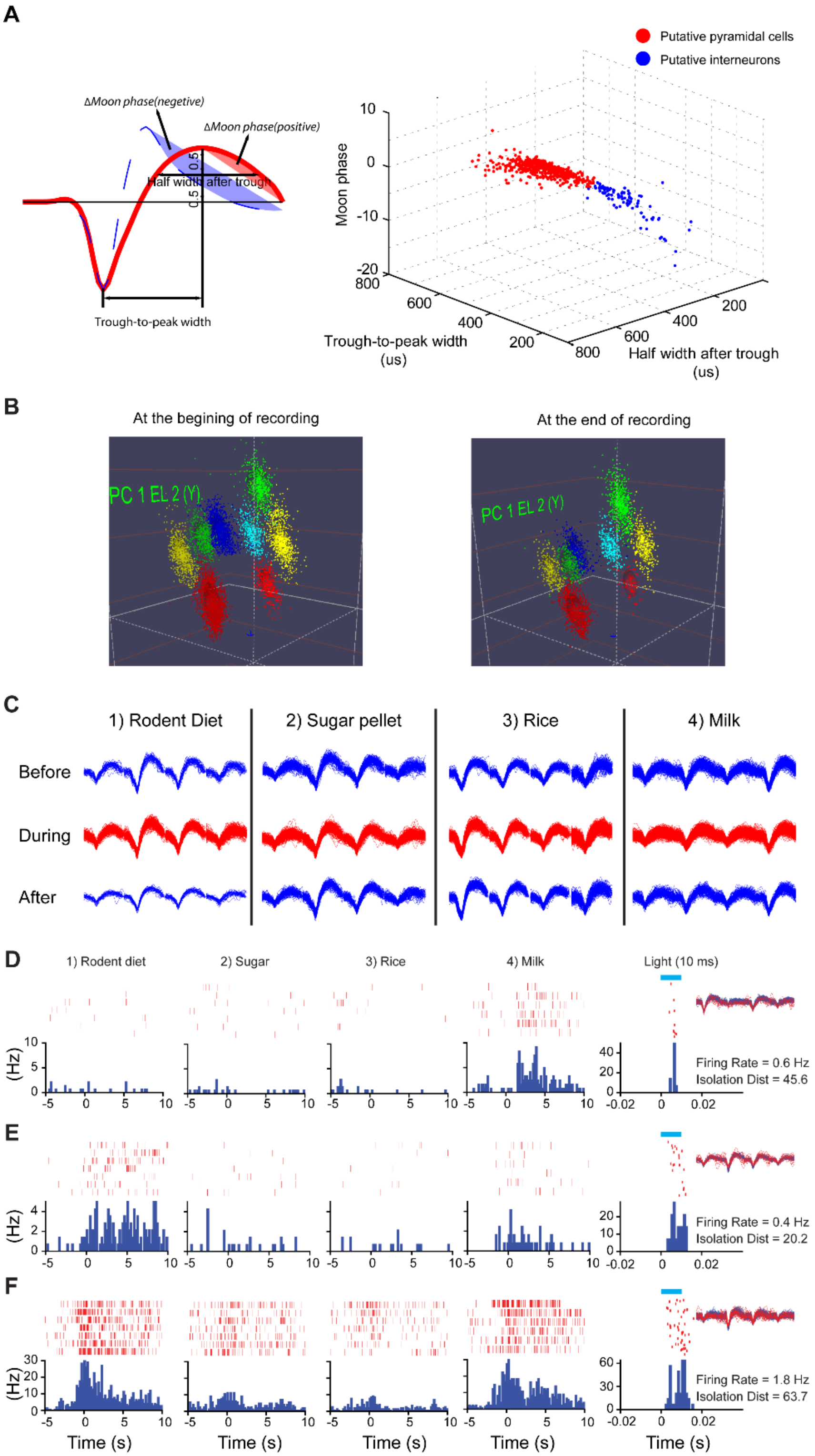

Suppl. Fig. 1. Classification of putative pyramidal cells and optogenetic confirmation of pyramidal cells.

(A) Classification of putative pyramidal units and interneurons in the BLA. **(B)** The stability of tetrode recordings. **(C)** Spike waveforms (voltage traces) were consistent before, during, and after food stimulation. To ensure that changes in firing patterns to foods were not simply due to electrode movement or waveform contamination upon consummatory behavior, we measured the recording stability and the waveforms of spikes before, during, and after food consumption. Our analysis showed that recordings were stable and the waveforms during stimulation were identical to those before and after the appetitive stimulation period. This indicates no contamination among spikes from environmental or motion-related noise. **(D)** A BLA pyramidal cell which was identified by blue light stimulation increased its firing specifically to drinking milk. Light-triggered tetrode waveforms (red traces) were identical and overlaid to the spontaneously generated waveforms (blue traces). **(E)** A BLA pyramidal cell which was identified by blue light stimulation increased its firing to eat rodent diet and milk. **(F)** A BLA pyramidal cell which was identified by blue light stimulation increased its firing to multiple types of food experiences. The quality of spike sorting was shown by isolation distance. The basal firing rates were also shown. Please note that the units identified from the same tetrodes which did not change their firings (data not shown), further indicating that appetitive stimulation-triggered responses were specific to these cognitive inputs.

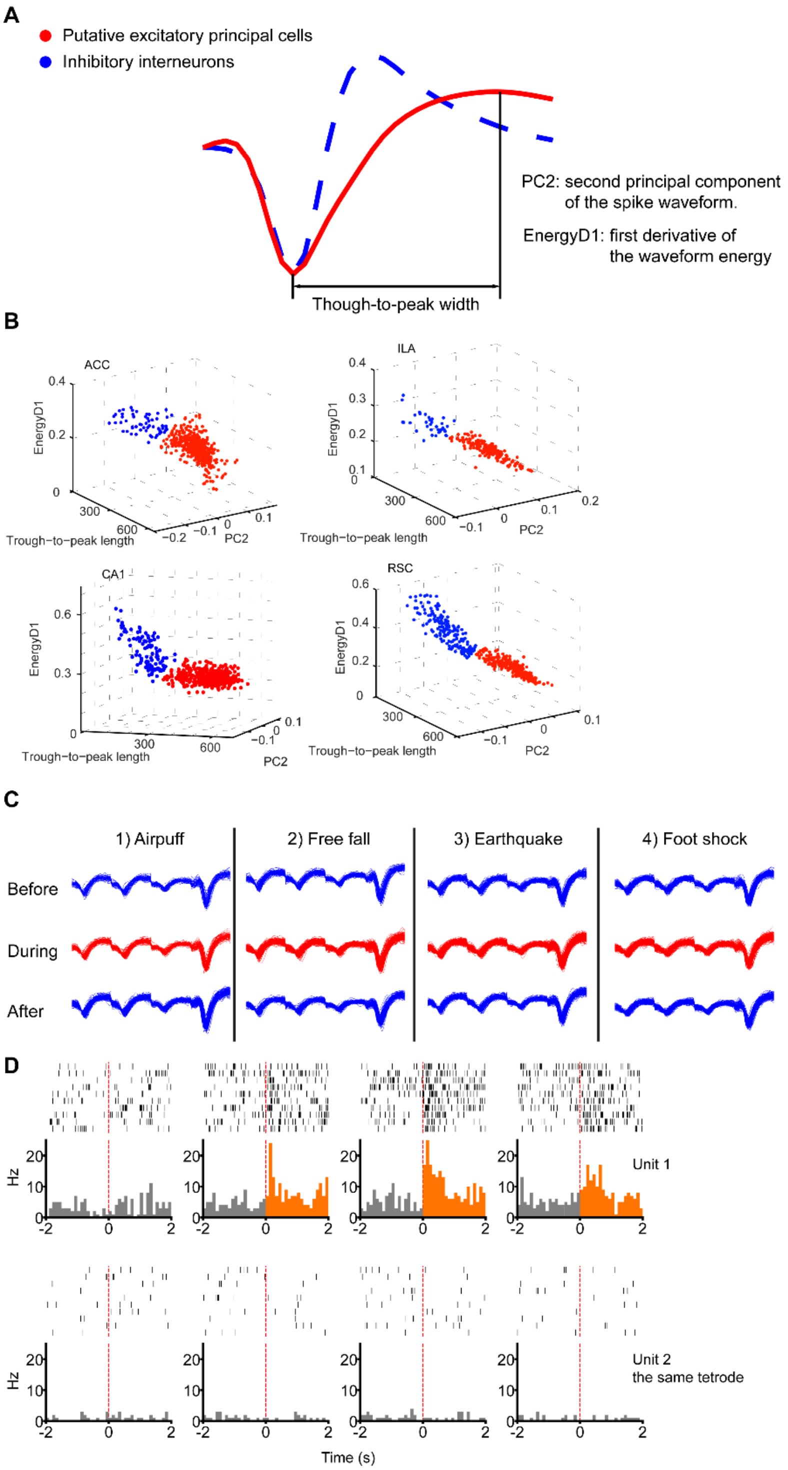

Suppl. Fig. 2. Classification of putative excitatory units and inhibitory neurons.

(A) Typical waveforms for principal units and interneurons. **(B)** Classification of ACC units, IL units, CA1 units, and RSC units. **(C)** Spike waveforms (voltage traces from tetrode's four channels) were assessed for their consistency before, during, and after fearful stimulation. **(D)** Two

example units recorded from the same tetrode, one unit increased its firings to three fearful stimuli (upper subpanels), the other unit did not change its firing (lower subpanels).

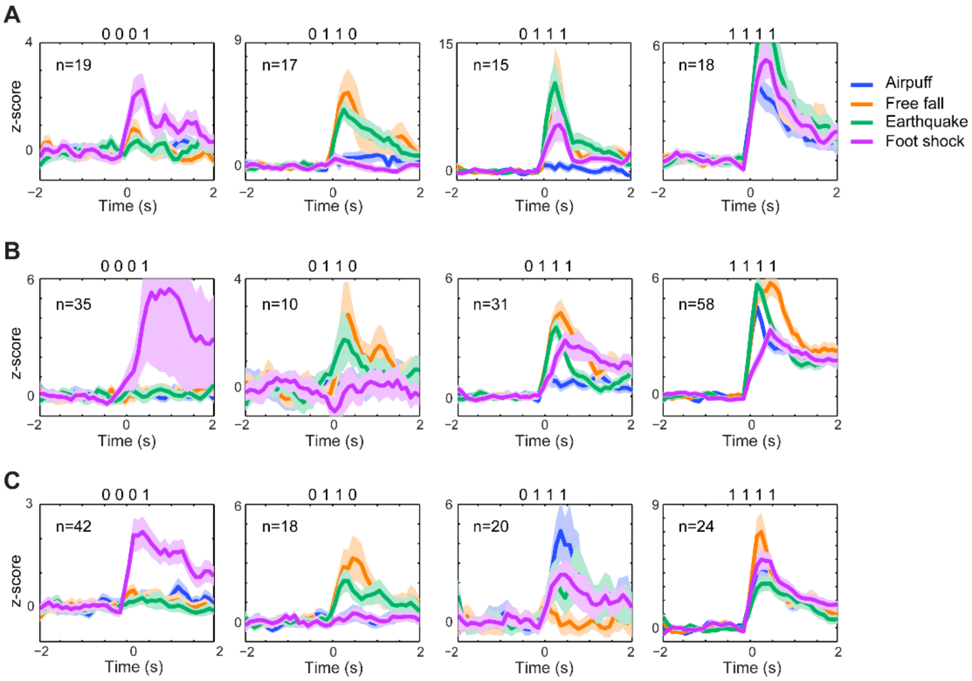

Suppl. Fig. 3. Population responses of distinct neural cliques in the IL, CA1 and RSC to fearful events. (A) The population responses of 1-event, 2-event, 3-event and 4-event neural cliques in the IL. **(B)** The population responses of 1-event, 2-event, 3-event and 4-event neural cliques in the CA1 region. **(C)** The population responses of 1-event, 2-event, 3-event and 4-event neural cliques in the RSC.

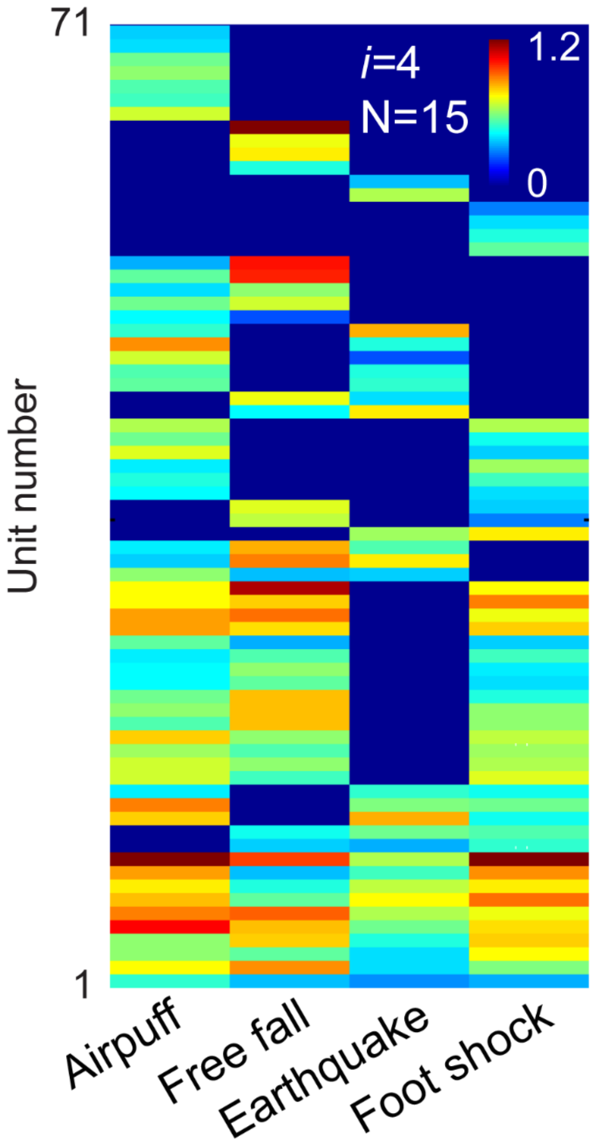

Suppl. Fig. 4. Specific-to-general 15 permutated neural cliques from the simultaneously recorded CA1 cells of a single mouse. Y-axis of the heat map lists the number of units, whereas X-axis labeled 1, 2, 3, and 4 corresponds to fearful stimuli air-puff, free-fall, earthquake, and foot-shock, respectively. Color scale bars indicate the logarithm transformed responsiveness of these units.

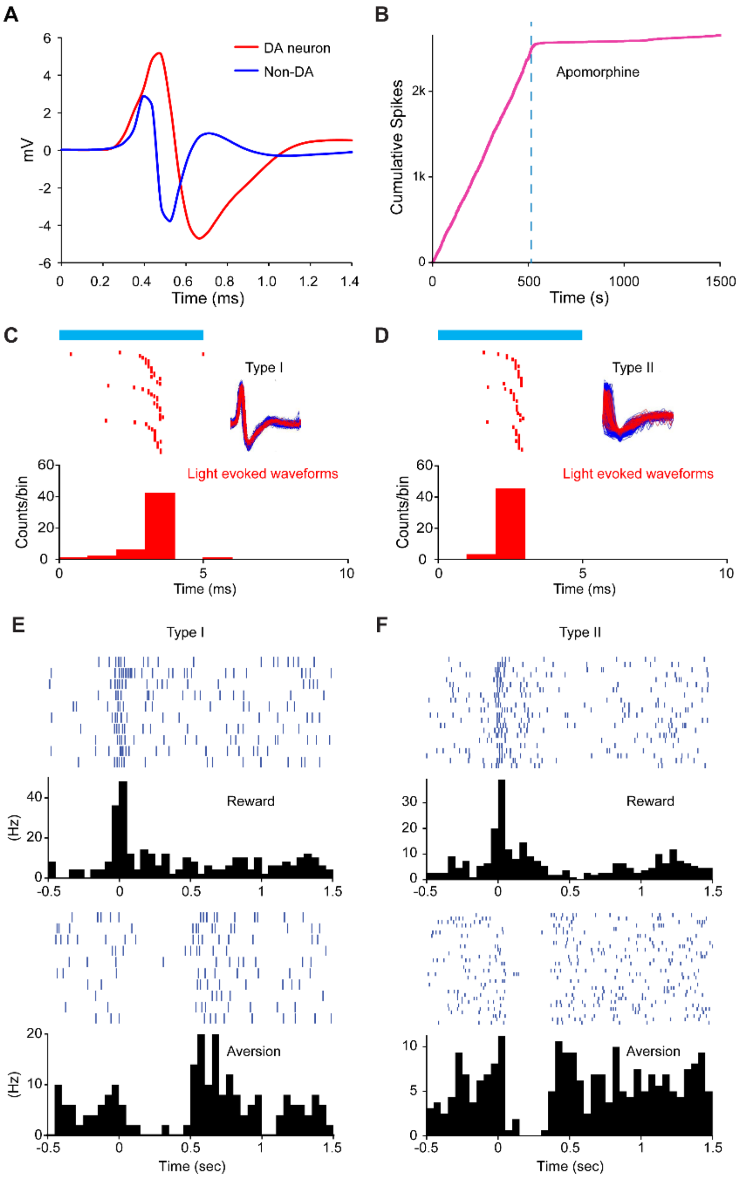

Suppl. Fig. 5. VTA DA neurons (type-1 and type-2) showed suppressed firings in response to fearful stimulations. (A) Waveforms of DA neurons and non-DA neurons. **(B)** Cumulative spike activity of a DA neuron in response to the apomorphine injection. **(C)** Optically evoked neural activity (red) of the type-1 DA neuron were identical to those of spontaneous waveforms. We found that blue light-stimulation evoked time-locked activation of such putative DA neurons, with the averaged time latencies at 4.4 ± 0.23 ms (Mean ± SEM, n = 4 units from three mice). **(D)** Optically evoked neural activity (red) of the type-2 DA neuron were identical to those of spontaneous waveforms. **(E)** The response of one representative type-1 DA neuron upon

responded to reward sugar pellets (upper panel) vs. aversive stimuli (0.5 s earthquake, lower panel). The earthquake induced suppression was followed by rebound excitation. **(F)** The response of type-2 DA neuron upon responded to reward sugar pellets (upper panel) vs. aversive stimuli (0.5 s earthquake, lower panel). Type-2 neurons did not have rebound excitation after the termination of earthquake.

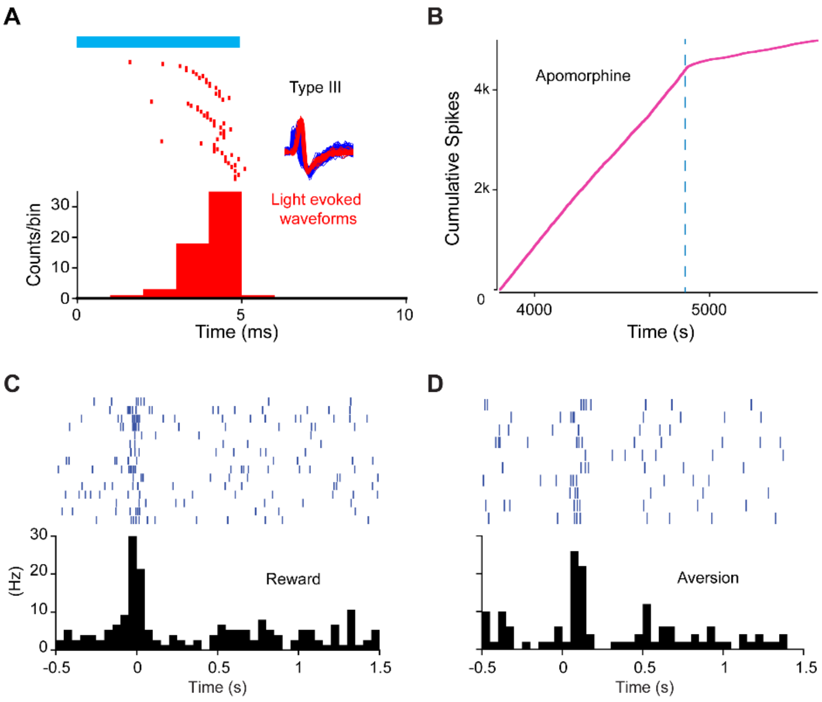

Suppl. Fig. 6. VTA DA neurons (type-3) showed increased firings in response to fearful stimulations. (A) Optically evoked neural activity (red) of the type-3 DA neuron were identical to those of spontaneous waveforms. **(B)** Cumulative spike activity of a type-3 DA neuron in response to the apomorphine injection. **(C)** The response of one representative type-3 DA neuron upon responded to reward sugar pellets. **(D)** The same unit also increased its firing upon aversive stimuli (0.5 s earthquake).
